# Perfluoroalkyl substances and changes in body weight and resting metabolic rate in response to weight-loss diets: A prospective study

**DOI:** 10.1371/journal.pmed.1002502

**Published:** 2018-02-13

**Authors:** Gang Liu, Klodian Dhana, Jeremy D. Furtado, Jennifer Rood, Geng Zong, Liming Liang, Lu Qi, George A. Bray, Lilian DeJonge, Brent Coull, Philippe Grandjean, Qi Sun

**Affiliations:** 1 Department of Nutrition, Harvard T.H. Chan School of Public Health, Boston, Massachusetts, United States of America; 2 Pennington Biomedical Research Center, Louisiana State University, Baton Rouge, Louisiana, United States of America; 3 Department of Epidemiology, Harvard T.H. Chan School of Public Health, Boston, Massachusetts, United States of America; 4 Department of Biostatistics, Harvard T.H. Chan School of Public Health, Boston, Massachusetts, United States of America; 5 Department of Epidemiology, School of Public Health and Tropical Medicine, Tulane University, New Orleans, Louisiana, United States of America; 6 Department of Environmental Health, Harvard T.H. Chan School of Public Health, Boston, Massachusetts, United States of America; 7 Institute of Public Health, University of Southern Denmark, Odense, Denmark; 8 Channing Division of Network Medicine, Department of Medicine, Brigham and Women’s Hospital and Harvard Medical School, Boston, Massachusetts, United States of America; Stanford University, UNITED STATES

## Abstract

**Background:**

The potential endocrine-disrupting effects of perfluoroalkyl substances (PFASs) have been demonstrated in animal studies, but whether PFASs may interfere with body weight regulation in humans is largely unknown. This study aimed to examine the associations of PFAS exposure with changes in body weight and resting metabolic rate (RMR) in a diet-induced weight-loss setting.

**Methods and findings:**

In the 2-year POUNDS Lost randomized clinical trial based in Boston, Massachusetts, and Baton Rouge, Louisiana, that examined the effects of energy-restricted diets on weight changes, baseline plasma concentrations of major PFASs were measured among 621 overweight and obese participants aged 30–70 years. Body weight was measured at baseline and 6, 12, 18, and 24 months. RMR and other metabolic parameters, including glucose, lipids, thyroid hormones, and leptin, were measured at baseline and 6 and 24 months. Participants lost an average of 6.4 kg of body weight during the first 6 months (weight-loss period) and subsequently regained an average of 2.7 kg of body weight during the period of 6–24 months (weight regain period). After multivariate adjustment, baseline PFAS concentrations were not significantly associated with concurrent body weight or weight loss during the first 6 months. In contrast, higher baseline levels of PFASs were significantly associated with a greater weight regain, primarily in women. In women, comparing the highest to the lowest tertiles of PFAS concentrations, the multivariate-adjusted mean weight regain (SE) was 4.0 (0.8) versus 2.1 (0.9) kg for perfluorooctanesulfonic acid (PFOS) (*P*_trend_ = 0.01); 4.3 (0.9) versus 2.2 (0.8) kg for perfluorooctanoic acid (PFOA) (*P*_trend_ = 0.007); 4.7 (0.9) versus 2.5 (0.9) kg for perfluorononanoic acid (PFNA) (*P*_trend_ = 0.006); 4.9 (0.9) versus 2.7 (0.8) kg for perfluorohexanesulfonic acid (PFHxS) (*P*_trend_ = 0.009); and 4.2 (0.8) versus 2.5 (0.9) kg for perfluorodecanoic acid (PFDA) (*P*_trend_ = 0.03). When further adjusted for changes in body weight or thyroid hormones during the first 6 months, results remained similar. Moreover, higher baseline plasma PFAS concentrations, especially for PFOS and PFNA, were significantly associated with greater decline in RMR during the weight-loss period and less increase in RMR during the weight regain period in both men and women. Limitations of the study include the possibility of unmeasured or residual confounding by socioeconomic and psychosocial factors, as well as possible relapse to the usual diet prior to randomization, which could have been rich in foods contaminated by PFASs through food packaging and also dense in energy.

**Conclusions:**

In this diet-induced weight-loss trial, higher baseline plasma PFAS concentrations were associated with a greater weight regain, especially in women, possibly explained by a slower regression of RMR levels. These data illustrate a potential novel pathway through which PFASs interfere with human body weight regulation and metabolism. The possible impact of environmental chemicals on the obesity epidemic therefore deserves attention.

**Trial registration:**

ClinicalTrials.gov NCT00072995

## Abstract 2

Gang Liu and colleagues reveal that perfluoroalkyl substances, often found in food packaging particularly of energy dense foods, contribute to weight regain for those recruited to a weight loss trial. This effect is higher in women.

## Author summary

**Why was this study done?:**

- Although many approaches can be used to achieve a short-term weight loss, maintenance of weight loss has become a key challenge for sustaining long-term benefits of weight loss. Accumulating evidence has suggested that certain environmental compounds may play an important role in weight gain and obesity development.
- The potential endocrine-disrupting effects of perfluoroalkyl substances (PFASs) have been demonstrated in animal studies, but whether PFASs may interfere with body weight regulation in humans is largely unknown.

**What did the researchers do and find?:**

- In a 2-year diet-induced weight-loss trial (the POUNDS Lost trial), we measured plasma concentrations of PFASs at baseline in 621 overweight and obese men and women and collected information on changes in body weight, resting metabolic rate (RMR), and other metabolic parameters during weight loss and weight regain over the 2 years the participants were on the study diet.
- Higher baseline levels of PFASs were significantly associated with a greater weight regain, primarily in women. On average, women in the highest tertile of PFAS concentrations regained 1.7–2.2 kg more body weight than women in the lowest tertile.
- Higher baseline plasma concentrations of PFASs, especially perfluorooctanesulfonic acid (PFOS) and perfluorononanoic acid (PFNA), were significantly associated with greater decline in RMR during the first 6 months and less increase in RMR during the period when participants on average regained weight (6–24 months).

**What do these findings mean?:**

- In this diet-induced weight-loss trial, higher baseline PFAS concentrations were associated with a greater weight regain, especially in women, possibly explained by a slower return of RMR levels. These data provide initial evidence suggesting that PFASs may interfere with human body weight regulation and counteract efforts to maintain weight loss in adults.

## Introduction

Obesity has become a worldwide public health concern [1,2]. Based on recent US data, the prevalence of obesity is 37.7% in adults and 17.0% in children and adolescents, with no sign of a reduction in the foreseeable future [3–5]. Although many approaches can be used to achieve short-term weight loss, its maintenance remains a key challenge [6,7]. Meanwhile, given the same intervention strategies, apparent within-group variability in weight loss and weight regain has been demonstrated [7,8]. Although the exact reasons for the variability are largely unknown, accumulating evidence has suggested that certain environmental compounds may play an important role in weight gain and obesity development [9,10].

Perfluoroalkyl substances (PFASs), especially perfluorooctanoic acid (PFOA) and perfluorooctanesulfonic acid (PFOS), have been identified as plausible endocrine disruptors with the potential to perturb weight regulation [9,11–14]. Evidence from animal studies has suggested that PFASs may be involved in altering energy metabolism and thyroid hormone homeostasis [15–17], likely through the activation of various transcriptional factors, such as the peroxisome proliferator-activated receptors (PPARs) [18–20]. However, given the species-specific toxicokinetics and tissue distribution of PFASs [18], extrapolation from animals to humans has yet to be supported. Although some human studies have examined the potential intergenerational effects of PFASs on body weight, the findings were somewhat inconsistent [21–27]. To our knowledge, no prospective study has explored the association between PFAS exposure and weight change in adults under controlled circumstances. Furthermore, it is largely unknown whether resting metabolic rate (RMR) or thyroid hormones, factors that can influence energy expenditure [28], might be also involved in the potential effects of PFASs on weight regulation [29,30].

PFASs are extensively used in many industrial and consumer products, including food packaging, paper and textile coatings, and non-stick cookware [31–34]. A recent study reported that the drinking water supplies for at least 6 million US citizens may exceed the US Environmental Protection Agency’s health advisory limit for lifetime exposure to PFOS and PFOA from drinking water [35]. In addition, these compounds are extremely stable in the environment and have a long elimination half-life in the human body [36], thus rendering PFASs a possible threat to human health. Due to the potential metabolic abnormalities associated with elevated PFAS levels, we aimed to examine the associations of PFAS exposure with changes in body weight and RMR in the well-designed and rigorously conducted POUNDS (Preventing Overweight Using Novel Dietary Strategies) Lost trial [37].

## Methods

### Ethics statement

The protocol was approved by the institutional review boards at the Harvard T.H. Chan School of Public Health, Brigham and Women’s Hospital, and the Pennington Biomedical Research Center of the Louisiana State University System, as well as by a data and safety monitoring board appointed by the National Heart, Lung, and Blood Institute. All participants provided written informed consent. The trial was registered at ClinicalTrials.gov (NCT00072995).

### Study participants

The POUNDS Lost study, a 2-year randomized clinical trial, was designed to compare the effects of 4 energy-reduced diets with different macronutrient (i.e., fat, protein, and carbohydrate) compositions on body weight, as previously described [37]. At baseline, 811 overweight and obese men and women aged 30–70 years were randomly assigned to 1 of 4 diets that consisted of different compositions of similar foods and met the guidelines for cardiovascular health. Eighty percent of the participants (*n =* 645) completed the trial. Each participant’s caloric prescription for the 2-year period represented a deficit of 750 kcal per day from baseline, as calculated from each individual’s resting energy expenditure and activity level [37]. All participants had normal thyroid function at study baseline [38]. The main findings of this trial were that most of the weight loss was observed in the first 6 months, followed by a gradual weight regain through to 24 months, and that the weight changes (both weight loss and weight regain) did not differ significantly between the diet groups [37].

The current analysis included 621 participants with available fasting plasma samples collected at baseline. Of these individuals, 592 and 460 participants also provided blood samples at 6 months and 2 years, respectively.

### Measurements of anthropometry and RMR

In the morning before breakfast and after urination, body weight and waist circumference were measured at baseline and 6, 12, 18, and 24 months. Body mass index (BMI) was calculated as body weight in kilograms divided by height in meters squared. At baseline and 6 and 24 months, body fat mass and lean mass (*n =* 424) were measured using dual energy X-ray absorptiometry (DXA) (Hologic QDR 4500A bone densitometer; Hologic); visceral and subcutaneous abdominal fat (*n =* 165) were measured using a computed tomography (CT) scanner [39]; and blood pressure was measured by an automated device (Omron HEM907XL; Omron). RMR was assessed at baseline and 6 and 24 months using a Deltatrac II Metabolic Monitor (Datex-Ohmeda) after an overnight fast [40]. Briefly, after a 30-minute rest, a transparent plastic hood was placed over the head of the participant for another 30 minutes. Participants were required to keep still and awake during the test, and the last 20 minutes of measurements were used for the calculation of RMR [40].

### Laboratory measurements of PFASs and other metabolic markers

Plasma concentrations of PFOS, PFOA, perfluorononanoic acid (PFNA), perfluorohexanesulfonic acid (PFHxS), and perfluorodecanoic acid (PFDA) were measured at baseline only, using a sensitive and reliable method based on online solid phase extraction and liquid chromatography coupled to a triple quadropole mass spectrometer [41], with minor modifications. Due to the long elimination half-lives of the PFASs and incomplete samplings, we did not measure plasma PFAS levels during the trial. For all major PFASs, the concentrations were above the limit of detection (0.05 ng/ml), and the inter- and intra-assay coefficients of variation were <6.3% and <6.1%, respectively.

In our pilot study evaluating the within-person stability of PFAS concentrations, intra-class correlation coefficients (ICCs) between concentrations in 2 blood samples collected 1–2 years apart from 58 participants in the Nurses’ Health Study II demonstrated excellent reproducibility of PFAS concentrations in blood: the ICCs were 0.91 for PFOS, 0.90 for PFOA, 0.94 for PFHxS, 0.87 for PFNA, and 0.82 for PFDA (all *P <* 0.001).

At baseline, 6 months, and 24 months, fasting plasma glucose, insulin, total cholesterol, high-density lipoprotein (HDL) cholesterol, low-density lipoprotein (LDL) cholesterol, and triglycerides were measured on the Synchron CX7 (Beckman Coulter), and hemoglobin A1C (HbA1c) was measured on a Synchron CX5 (Beckman Coulter); plasma leptin and soluble leptin receptor were measured by an ultrasensitive immunoassay (R&D Systems); and serum free triiodothyronine (T3), free thyroxine (T4), total T3, total T4, and thyroid stimulating hormone were measured using a competitive electrochemiluminescence immunoassay on the Roche E modular system (Roche Diagnostics), as previously described elsewhere [37]. The homeostatic model assessment of insulin resistance (HOMA-IR) value was calculated using the updated HOMA model (HOMA2) described by Levy et al. [42]. Adipose tissue was obtained from 103 participants at baseline and at 6 months. Gene expression was measured by direct hybridization using the Illumina HumanHT-12 v3 Expression BeadChip (Illumina) (details in S1 Text).

### Assessments of other covariates

Using standardized questionnaires, we obtained information on age, sex, race (white, black, Hispanic, or other), educational attainment (high school or less, some college, or college graduate or beyond), smoking status (never, former, or current smoker), alcohol consumption (drinks/week), menopausal status (yes or no), and hormone replacement therapy use (yes or no). At baseline, 6 months, and 24 months, physical activity was assessed using the Baecke physical activity questionnaire, which included 16 items inquiring about levels of habitual physical activities (i.e., physical activity at work, sports during leisure time, and other physical activity during leisure time) [43]. All responses were pre-coded on 5-point scales. Total physical activity was expressed as the average of the sum of the individual responses, with a score ranging from 0 to 5 [43].

### Statistical analysis

The comparisons between participants included in the current analysis and those excluded were evaluated by the Student’s *t* test for normally distributed variables, the Wilcoxon rank-sum test for skewed variables, and the chi-squared test for categorical variables. The associations between baseline PFASs and changes in body weight and RMR during the period of weight loss (first 6 months) or weight regain (6–24 months) were examined using linear regression. The least-square means of changes in body weight (at 6, 12, 18, and 24 months) and RMR (at 6 and 24 months) according to tertiles of baseline PFAS concentrations were calculated. In addition, the relationship between PFASs and other potential mediators including thyroid hormones and leptin were further evaluated using linear regression. Covariates considered in multivariate adjustments included baseline age (continuous), sex, race, educational attainment (high school or less, some college, or college graduate or beyond), smoking status (never, former, or current smoker), alcohol consumption (continuous), physical activity (continuous), the 4 diet groups, and baseline BMI (or baseline RMR for the analysis of RMR change). Moreover, menopausal status and hormone replacement therapy (women only) were also entered into the model in a sensitivity analysis. To test the linear trend of the associations of baseline PFAS concentrations with changes in body weight and RMR, we assigned a median value to each tertile of PFAS concentration and treated it as a continuous variable. We also tested the linear trend using the PFAS concentrations as continuous variables (log_10_-transformed). In an exploratory analysis, factor analysis was used to explore the potential exposure patterns of PFASs.

To investigate the associations of baseline PFASs with baseline values of and changes in other metabolic parameters (including glucose, lipids, thyroid hormones, and leptin), Spearman correlation coefficients (*r*_s_) were calculated with adjustment for the potential confounders mentioned above. Stratified analysis was also conducted according to sex, and a likelihood ratio test was performed to test for potential interactions. In sensitivity analyses, body weight or RMR at 6 months (or changes during the first 6 months), instead of the baseline value, was included in the multivariate models when examining the associations between baseline PFASs and changes in body weight or RMR during the period of 6–24 months. We also stratified the analyses by dietary intervention group. In addition, to account for the correlations between measurements on the same individuals, linear mixed-effects models were also used to examine the associations between baseline PFAS concentrations and weight regain (weight measurements at 6, 12, 18, and 24 months), with an unstructured covariance matrix. To assess confounding patterns, in another sensitivity analysis, the covariates were entered into the model in a stepwise manner. In an exploratory analysis, we also examined the associations of PFAS exposures with the gene expression profile in adipose tissue (S1 Text).

A 2-sided *P* < 0.05 was considered statistically significant. The statistical analyses were performed with SAS software, version 9.4 (SAS Institute).

## Results

### Study population

The mean (SD) age of the 621 participants was 51.4 (9.1) years, with a mean (SD) baseline BMI of 32.6 (3.8) kg/m^2^. Participants lost an average of 6.4 kg of body weight during the first 6 months and subsequently regained an average of 2.7 kg during the remaining study period. In comparison with the POUNDS Lost participants not included in the current study due to the lack of plasma samples at baseline, the participants included were slightly older (51.4 versus 49.1 years, *P* = 0.01), but there were no significant differences in other characteristics, including body weight and RMR (S1 Table).

### Associations between PFASs, body weight, and other metabolic parameters at baseline

Table 1 shows the baseline characteristics of the study participants. PFOS and PFOA were the dominant PFASs. The median (interquartile range) plasma concentration was 24.5 (16.2–37.0) ng/ml for PFOS, 4.5 (3.3–6.3) ng/ml for PFOA, 2.4 (1.5–3.6) ng/ml for PFHxS, 1.5 (1.0–2.4) ng/ml for PFNA, and 0.37 (0.27–0.52) ng/ml for PFDA. At baseline, significant inter-correlations were observed between PFOS, PFOA, PFHxS, PFNA, and PFDA (*r*_s_ ranged from 0.38 to 0.85) (S2 Table), although no particular pattern of PFAS mixture was identified in the factor analysis. After multivariate adjustment, PFOS, PFOA, and PFNA concentration were all positively associated with insulin, HOMA-IR, diastolic blood pressure, and free T3 (*r*_s_ ranged from 0.10 to 0.18, all *P <* 0.05) at baseline. In addition, certain PFASs (e.g., PFHxS and PFDA) were positively associated with some of the variables, including visceral fat mass, systolic blood pressure, glucose, triglycerides, LDL cholesterol, free T4, total T4, and leptin (*r*_s_ ranged from 0.08 to 0.24, all *P <* 0.05) (S2 Table). No PFASs were correlated with body weight, waist circumference, BMI, or RMR at baseline.

**Table 1:** Baseline characteristics of participating men and women.

Characteristic	Men (*n =* 237)	Women (*n =* 384)	*P*-value^a^
Age (years)	51.9 ± 9.6	51.1 ± 8.8	0.26
Race white	89.9%	74.7%	<0.001
BMI (kg/m^2^)	33.4 ± 3.4	32.0 ± 3.9	<0.001
Weight (kg)	104.1 ± 12.7	85.7 ± 12.6	<0.001
Waist circumference (cm)	113.2 ± 10.1	97.2 ± 10.9	<0.001
Resting metabolic rate (kcal/24 h)	1,821.9 ± 243.6	1,378.6 ± 184.6	<0.001
Education level			0.19
High school or less	11.0%	9.0%	
Some college	17.3%	23.2%	
College graduate or beyond	71.7%	61.8%	
Smoking status			0.006
Never smoked	51.9%	64.9%	
Past smoker	43.9%	32.0%	
Current smoker	4.2%	3.1%	
Alcohol consumption (drinks/week)	2.0 (0–5.0)	1.0 (0–2.0)	<0.001
Physical activity^b^	1.60 ± 0.1	1.56 ± 0.1	
Systolic blood pressure (mm Hg)	124.0 ± 12.2	117.0 ± 13.7	<0.001
Diastolic blood pressure (mm Hg)	77.7 ± 9.1	74.0 ± 8.9	<0.001
Glucose (mmol/l)	5.2 (4.9–5.5)	4.9 (4.6–5.1)	<0.001
Insulin (pmol/l)^c^	84.7 (61.1–117.4)	63.9 (45.1–101.4)	<0.001
Total cholesterol (mmol/l)	5.9 (4.8–8.5)	5.6 (4.8–6.8)	0.002
LDL cholesterol (mmol/l)	3.1 (2.6–3.7)	3.3 (2.8–3.8)	0.003
HDL cholesterol (mmol/l)	1.0 (0.9–1.2)	1.3 (1.1–1.6)	<0.001
Triglycerides (mmol/l)	2.0 ± 1.1	1.4 ± 0.8	<0.001
Free T3 (pmol/l)^d^	5.2 (4.8–5.5)	4.8 (4.4–5.1)	<0.001
Free T4 (pmol/l)^d^	15.3 (14.1–16.2)	14.8 (13.5–16.2)	0.002
PFOS (ng/ml)	27.2 (19.9–45.2)	22.3 (14.3–34.9)	<0.001
PFOA (ng/ml)	5.2 (3.9–6.8)	4.1 (2.8–5.6)	<0.001
PFHxS (ng/ml)	3.1 (2.3–4.4)	1.9 (1.2–3.0)	<0.001
PFNA (ng/ml)	1.6 (1.1–2.4)	1.5 (1.0–2.4)	0.07
PFDA (ng/ml)	0.4 (0.3–0.5)	0.4 (0.3–0.6)	0.38

Data are mean ± SD, median (interquartile range), or percentage.

^a^The comparisons were examined using the Student’s *t* test for normally distributed variables, the Wilcoxon rank-sum test for skewed variables, and the chi-squared test for categorical variables.

^b^Physical activity was estimated by the Baecke questionnaire.

^c^One participant had missing value for insulin.

^d^In all, 30 men and 60 women had missing values for free T3 and free T4.

HDL, high-density lipoprotein; LDL, low-density lipoprotein; PFDA, perfluorodecanoic acid; PFHxS, perfluorohexanesulfonic acid; PFNA, perfluorononanoic acid; PFOA, perfluorooctanoic acid; PFOS, perfluorooctanesulfonic acid; T3, triiodothyronine; T4, thyroxine.

### Baseline PFASs and body weight changes

After multivariate adjustment including smoking status, physical activity, baseline BMI, and dietary intervention group, baseline PFAS concentrations were not associated with weight loss in the first 6 months (Table 2). The crude positive associations between certain PFAS levels and weight loss were abolished after multivariate adjustment (Table 2). In contrast, after multivariate adjustment, baseline PFOS and PFNA concentrations were positively associated with greater weight regain in the total study population. Comparing the highest to the lowest tertiles, the least-square means (SEs) of weight regain were 3.3 (0.6) versus 1.8 (0.6) kg for PFOS (*P*_trend_ = 0.009) and 3.4 (0.6) versus 2.0 (0.6) kg for PFNA (*P*_trend_ = 0.01) (Model 2 in Table 2). The results were similar when PFAS concentrations were treated as continuous variables (the beta coefficients for per-unit log_10_-transformed PFOS and PFNA increment were 0.80 and 1.02, respectively; both *P*_continuous_ < 0.05) (Table 2). After further adjusting for baseline thyroid hormones (Model 3 in Table 2), the associations remained significant. In sensitivity analyses, when body weight at baseline or 6 months (instead of BMI at baseline) was adjusted for in the models, the results were largely unchanged. When changes in body weight or changes in thyroid hormones or leptin during the first 6 months were also included as covariates, the results did not change materially. In addition, similar results were obtained when using linear mixed-effects models. When PFAS levels were categorized into quartiles, the results were largely similar.

**Table 2:** Changes in body weight^a^ according to tertiles of PFAS concentrations.

PFAS	Tertile of PFAS concentration (ng/ml)			*P*_trend_	*P*_continuous_^b^
	Tertile 1	Tertile 2	Tertile 3		
**Weight change (kg) during first 6 months; total *n =* 621**					
**PFOS**	<19.2	19.2–32.1	>32.1		
Median (IQR)	13.3 (9.7–16.2)	24.4 (21.4–28.5)	47.1 (37.0–58.8)		
Model 1 (unadjusted)	−5.2 ± 0.4	−7.1 ± 0.4	−7.0 ± 0.4	0.01	0.02
Model 2	−4.7 ± 0.6	−5.5 ± 0.6	−5.3 ± 0.6	0.27	0.99
Model 3	−5.0 ± 0.6	−5.7 ± 0.6	−5.3 ± 0.6	0.63	0.59
**PFOA**	<3.7	3.7–5.5	>5.5		
Median (IQR)	2.8 (2.2–3.4)	4.5 (4.1–4.9)	7.1 (6.3–8.3)		
Model 1 (unadjusted)	−5.7 ± 0.4	−6.6 ± 0.4	−6.9 ± 0.4	0.05	0.02
Model 2	−5.3 ± 0.6	−5.1 ± 0.6	−5.1 ± 0.6	0.73	0.70
Model 3	−5.5 ± 0.6	−5.4 ± 0.6	−5.0 ± 0.6	0.41	0.39
**PFHxS**	<1.8	1.8–3.1	>3.1		
Median (IQR)	1.2 (0.8–1.5)	2.4 (2.1–2.7)	4.3 (3.6–5.5)		
Model 1 (unadjusted)	−5.7 ± 0.4	−6.5 ± 0.4	−7.0 ± 0.4	0.01	0.02
Model 2	−5.3 ± 0.6	−5.1 ± 0.6	−5.1 ± 0.6	0.76	0.45
Model 3	−5.6 ± 0.6	−5.1 ± 0.6	−5.2 ± 0.7	0.54	0.22
**PFNA**	<1.1	1.1–2.0	>2.0		
Median (IQR)	0.89 (0.74–1.0)	1.5 (1.3–1.7)	3.0 (2.4–4.0)		
Model 1 (unadjusted)	−5.3 ± 0.4	−6.9 ± 0.4	−7.0 ± 0.4	0.01	0.01
Model 2	−4.7 ± 0.6	−5.4 ± 0.6	−5.4 ± 0.6	0.18	0.29
Model 3	−5.0 ± 0.6	−5.5 ± 0.6	−5.5 ± 0.6	0.35	0.54
**PFDA**	<0.31	0.31–0.47	>0.47		
Median (IQR)	0.24 (0.19–0.28)	0.37 (0.34–0.42)	0.61 (0.52–0.75)		
Model 1 (unadjusted)	−6.0 ± 0.4	−6.4 ± 0.4	−6.8 ± 0.4	0.18	0.21
Model 2	−5.0 ± 0.6	−5.1 ± 0.6	−5.4 ± 0.6	0.49	0.45
Model 3	−5.2 ± 0.6	−5.3 ± 0.6	−5.5 ± 0.6	0.56	0.76
**Weight change (kg) during 6–24 months; total *n =* 520**					
**PFOS**					
Model 1 (unadjusted)	1.6 ± 0.4	3.3 ± 0.4	3.2 ± 0.4	0.003	0.01
Model 2	1.8 ± 0.6	3.4 ± 0.6	3.3 ± 0.6	0.009	0.03
Model 3	1.5 ± 0.6	3.5 ± 0.6	3.2 ± 0.6	0.007	0.02
**PFOA**					
Model 1 (unadjusted)	1.8 ± 0.4	3.3 ± 0.4	3.0 ± 0.4	0.03	0.04
Model 2	2.2 ± 0.6	3.6 ± 0.6	3.0 ± 0.6	0.16	0.12
Model 3	1.8 ± 0.6	3.6 ± 0.6	2.9 ± 0.6	0.07	0.06
**PFHxS**					
Model 1 (unadjusted)	2.4 ± 0.4	2.5 ± 0.4	3.1 ± 0.4	0.26	0.42
Model 2	2.6 ± 0.6	2.7 ± 0.6	3.2 ± 0.6	0.32	0.49
Model 3	2.4 ± 0.6	2.7 ± 0.6	3.3 ± 0.7	0.18	0.21
**PFNA**					
Model 1 (unadjusted)	1.7 ± 0.4	2.9 ± 0.4	3.5 ± 0.4	<0.001	0.001
Model 2	2.0 ± 0.6	3.1 ± 0.6	3.4 ± 0.6	0.01	0.01
Model 3	1.8 ± 0.6	3.3 ± 0.6	3.5 ± 0.6	0.007	0.008
**PFDA**					
Model 1 (unadjusted)	2.1 ± 0.4	2.9 ± 0.4	3.1 ± 0.4	0.05	0.06
Model 2	2.3 ± 0.6	3.0 ± 0.6	3.1 ± 0.6	0.16	0.14
Model 3	2.0 ± 0.6	3.0 ± 0.6	3.2 ± 0.6	0.05	0.06

Model 1, unadjusted; Model 2, adjusted for age, sex, race, baseline BMI, education, smoking status, alcohol consumption, physical activity, and dietary intervention group; Model 3, further adjusted for baseline free T3 and free T4 levels.

^a^Data are least-square means ± standard errors calculated from general linear models.

^b^PFAS levels were log_10_-transformed before analysis.

PFAS, perfluoroalkyl substance; PFDA, perfluorodecanoic acid; PFHxS, perfluorohexanesulfonic acid; PFNA, perfluorononanoic acid; PFOA, perfluorooctanoic acid; PFOS, perfluorooctanesulfonic acid; T3, triiodothyronine; T4, thyroxine.

### Sex-specific associations between PFASs and weight regain

**Fig 1:**
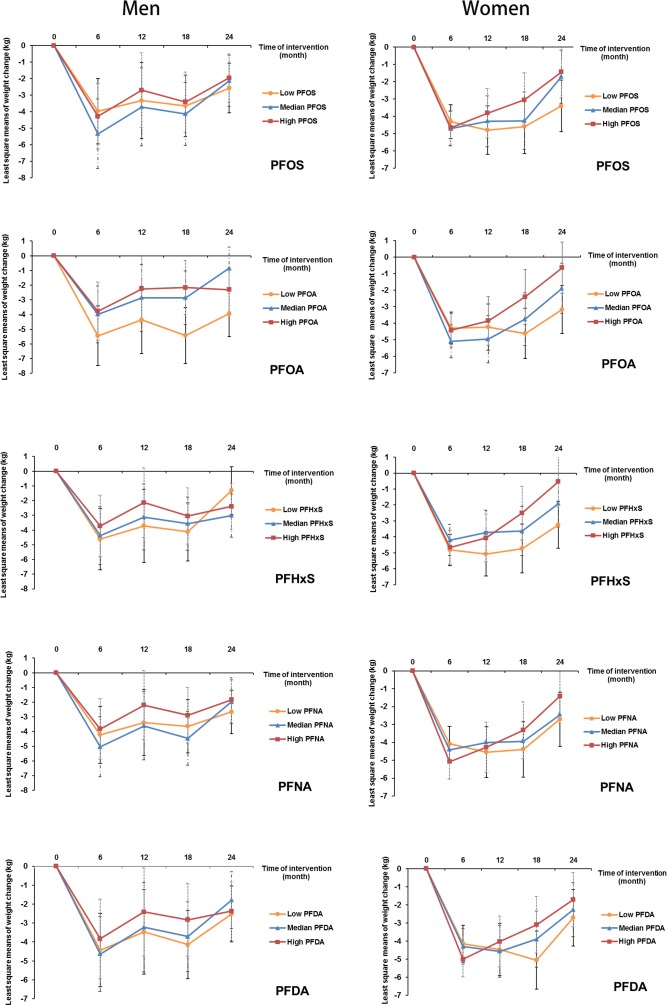
Trajectory of changes in body weight in men and women according to tertiles of PFAS concentrations. Data are least-square means, adjusted for age, race, education, smoking, alcohol consumption, physical activity, menopausal status (women only), hormone replacement therapy (women only), dietary intervention group, baseline free T3 and free T4 levels, and baseline BMI. PFAS, perfluoroalkyl substance; PFDA, perfluorodecanoic acid; PFHxS, perfluorohexanesulfonic acid; PFNA, perfluorononanoic acid; PFOA, perfluorooctanoic acid; PFOS, perfluorooctanesulfonic acid; T3, triiodothyronine; T4, thyroxine.

In an analysis stratified by sex, significant associations with weight regain were observed for all individual PFASs in women, but not in men. Comparing the highest to the lowest tertiles, the least-square means (SEs) of weight regain in women were 4.0 (0.8) versus 2.1 (0.9) kg for PFOS (*P*_trend_ = 0.01); 4.3 (0.9) versus 2.2 (0.8) kg for PFOA (*P*_trend_ = 0.007); 4.9 (0.9) versus 2.7 (0.8) kg for PFHxS (*P*_trend_ = 0.009); 4.7 (0.9) versus 2.5 (0.9) kg for PFNA (*P*_trend_ = 0.006); and 4.2 (0.8) versus 2.5 (0.9) kg for PFDA (*P*_trend_ = 0.03) (Table 3). Significant interactions with sex were demonstrated for PFOA and PFHxS (*P*_interaction_ = 0.04 and 0.01, respectively). When the covariates were entered into the model in a stepwise manner, these results did not change materially (S3 Table). The trajectory of changes in body weight in men and women according to tertiles of PFAS concentrations is shown in Fig 1. The trajectory of changes in body weight among total participants is shown in S1 Fig.

**Table 3:** Sex-stratified analyses of changes in body weight according to baseline plasma PFAS concentrations.

PFAS	Sex	Tertile of PFAS concentration^a^			*P*_trend_	*P*_interaction_
		Tertile 1	Tertile 2	Tertile 3		
**Weight change (kg) during first 6 months**						
**PFOS**	Men	−4.7 ± 1.3	−6.2 ± 1.3	−5.1 ± 1.2	0.91	0.61
	Women	−4.7 ± 0.7	−5.2 ± 0.7	−5.1 ± 0.7	0.49	
**PFOA**	Men	−6.2 ± 1.3	−4.9 ± 1.2	−4.7 ± 1.2	0.26	0.34
	Women	−4.7 ± 0.7	−5.5 ± 0.7	−4.9 ± 0.7	0.65	
**PFHxS**	Men	−5.4 ± 1.4	−5.3 ± 1.2	−4.8 ± 1.3	0.56	0.84
	Women	−5.1 ± 0.7	−4.6 ± 0.7	−5.1 ± 0.8	0.85	
**PFNA**	Men	−5.0 ± 1.2	−6.0 ± 1.2	−4.7 ± 1.2	0.76	0.73
	Women	−4.4 ± 0.7	−4.8 ± 0.7	−5.5 ± 0.7	0.10	
**PFDA**	Men	−5.2 ± 1.2	−5.5 ± 1.2	−4.8 ± 1.3	0.70	0.51
	Women	−4.6 ± 0.8	−4.6 ± 0.7	−5.5 ± 0.7	0.16	
**Weight change (kg) during 6–24 months**						
**PFOS**	Men	1.2 ± 1.1	3.4 ± 1.2	2.5 ± 1.1	0.34	0.50
	Women	2.1 ± 0.9	4.1 ± 0.9	4.0 ± 0.8	0.01	
**PFOA**	Men	1.6 ± 1.1	3.1 ± 1.1	1.6 ± 1.1	0.78	0.04
	Women	2.2 ± 0.8	4.2 ± 0.9	4.3 ± 0.9	0.007	
**PFHxS**	Men	3.5 ± 1.2	1.5 ± 1.1	1.4 ± 1.1	0.17	0.01
	Women	2.7 ± 0.8	3.6 ± 0.9	4.9 ± 0.9	0.009	
**PFNA**	Men	1.4 ± 1.1	3.4 ± 1.1	2.2 ± 1.1	0.48	0.31
	Women	2.5 ± 0.9	2.9 ± 0.9	4.7 ± 0.9	0.006	
**PFDA**	Men	1.6 ± 1.1	3.2 ± 1.1	1.8 ± 1.2	0.75	0.33
	Women	2.5 ± 0.9	3.1 ± 0.9	4.2 ± 0.8	0.03	

Data are least-square means ± standard errors calculated from general linear models, with adjustment for age, race, baseline BMI, education, smoking status, alcohol consumption, physical activity, dietary intervention group, and baseline free T3 and free T4 levels. Participants included 237 men and 384 women in the first 6 months, and 202 men and 318 women during the period of 6–24 months.

^a^The cutoffs for each PFAS were the same as those in Table 2.

PFAS, perfluoroalkyl substance; PFDA, perfluorodecanoic acid; PFHxS, perfluorohexanesulfonic acid; PFNA, perfluorononanoic acid; PFOA, perfluorooctanoic acid; PFOS, perfluorooctanesulfonic acid; T3, triiodothyronine; T4, thyroxine.

### Baseline PFASs and changes in RMR

**Fig 2:**
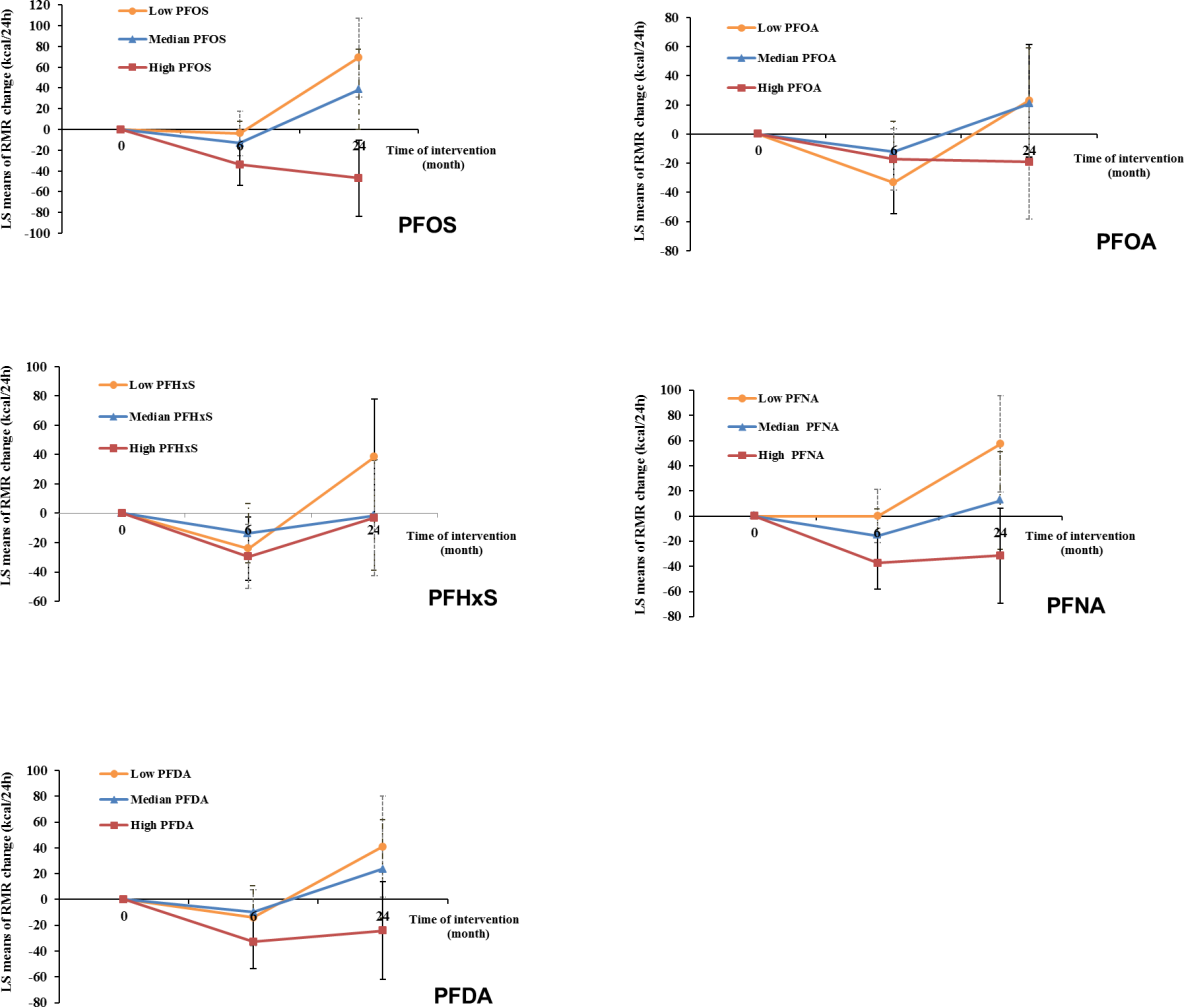
Trajectory of changes in RMR of all participants according to tertiles of PFAS concentrations. Data were adjusted for age, sex, race, education, smoking, alcohol consumption, physical activity, menopausal status (women only), hormone replacement therapy (women only), dietary intervention group, baseline free T3 and free T4 levels, and baseline RMR. LS, least-square; PFAS, perfluoroalkyl substance; PFDA, perfluorodecanoic acid; PFHxS, perfluorohexanesulfonic acid; PFNA, perfluorononanoic acid; PFOA, perfluorooctanoic acid; PFOS, perfluorooctanesulfonic acid; RMR, resting metabolic rate; T3, triiodothyronine; T4, thyroxine.

After multivariate adjustment, including baseline RMR and dietary intervention group, baseline plasma PFAS concentrations, especially for PFOS and PFNA, were significantly associated with a greater decline in RMR during the weight-loss period (first 6 months) and a lower increase in RMR during the weight regain period (6–24 months). During the first 6 months, comparing the highest to the lowest tertiles, the least-square means (SEs) of RMR change were −45.4 (15.5) versus −5.0 (16.3) kcal/day for PFOS (*P*_trend_ = 0.005) and −49.8 (15.9) versus −3.3 (16.1) kcal/day for PFNA (*P*_trend_ = 0.002) (Model 3 in Table 4). During the period of 6–24 months, comparing the highest to the lowest tertiles, the least-square means (SEs) of RMR change were 0.9 (26.2) versus 94.6 (27.5) kcal/day for PFOS (*P*_trend_ < 0.001); 12.7 (28.1) versus 69.3 (27.3) kcal/day for PFOA (*P*_trend_ = 0.03); 24.6 (28.5) versus 81.5 (27.5) kcal/day for PFHxS (*P*_trend_ = 0.03); 14.1 (27.7) versus 73.7 (27.6) kcal/day for PFNA (*P*_trend_ = 0.02); and 23.1 (27.6) versus 66.5 (28.2) kcal/day for PFDA (*P*_trend_ = 0.09) (Model 3 in Table 4). The results were similar when PFAS concentrations were treated as continuous variables (Table 4). When adjusting for RMR at 6 months (instead of RMR at baseline), the results maintained statistical significance. When changes in RMR or changes in thyroid hormones during the first 6 months were further adjusted for, the results remained largely unchanged. In the sex-stratified analysis, similar results were observed, although some associations did not reach statistical significance, possibly due to diminished power (S4 Table). No interaction between PFASs and sex on RMR changes was detected. The trajectory of changes in RMR among total participants according to tertiles of PFAS concentrations is shown in Fig 2. In addition, similar results were demonstrated when analyses were stratified by dietary intervention group.

**Table 4:** Changes in resting metabolic rate (RMR)^a^ according to tertiles of PFAS levels at baseline.

PFAS	Tertile of PFAS concentration			*P*_trend_	*P*_continuous_^b^
	T1	T2	T3		
**RMR change (kcal/day) during first 6 months; total *n =* 556**					
**PFOS**					
Model 1 (unadjusted)	−39.1 ± 10.4	−79.5 ± 10.2	−99.3 ± 10.1	<0.001	<0.001
Model 2	6.9 ± 15.6	−21.3 ± 15.7	−48.6 ± 14.8	<0.001	<0.001
Model 3	−5.0 ± 16.3	−24.7 ± 16.5	−45.4 ± 15.5	0.005	0.02
**PFOA**					
Model 1 (unadjusted)	−58.9 ± 10.4	−67.0 ± 10.2	−93.8 ± 10.4	0.02	0.03
Model 2	−21.4 ± 15.5	−18.2 ± 15.9	−31.3 ± 15.5	0.48	0.37
Model 3	−33.8 ± 16.2	−21.7 ± 16.4	−26.2 ± 16.2	0.61	0.86
**PFHxS**					
Model 1 (unadjusted)	−47.6 ± 10.4	−67.8 ± 10.1	−103.7 ± 10.3	<0.001	0.005
Model 2	−11.9 ± 15.4	−22.4 ± 15.5	−42.3 ± 16.0	0.04	0.20
Model 3	−23.3 ± 16.1	−21.6 ± 15.9	−41.0 ± 16.8	0.25	0.75
**PFNA**					
Model 1 (unadjusted)	−33.3 ± 10.3	−77.0 ± 10.0	−108.2 ± 10.2	<0.001	<0.001
Model 2	8.1 ± 15.3	−24.3 ± 15.4	−54.6 ± 15.1	<0.001	<0.001
Model 3	−3.3 ± 16.1	−27.5 ± 16.2	−49.8 ± 15.9	0.002	0.003
**PFDA**					
Model 1 (unadjusted)	−64.2 ± 10.5	−60.4 ± 10.2	−95.2 ± 10.4	0.03	0.01
Model 2	−8.8 ± 15.7	−15.7 ± 15.5	−43.1 ± 15.2	0.01	0.002
Model 3	−17.5 ± 16.6	−19.4 ± 15.9	−42.1 ± 15.8	0.09	0.05
**RMR change (kcal/day) during 6–24 months; total *n =* 393**					
**PFOS**					
Model 1 (unadjusted)	102.6 ± 16.5	82.5 ± 16.3	16.9 ± 16.6	<0.001	<0.001
Model 2	108.3 ± 27.4	83.3 ± 28.1	17.2 ± 26.0	<0.001	<0.001
Model 3	94.6 ± 27.5	67.3 ± 28.3	0.9 ± 26.2	<0.001	0.001
**PFOA**					
Model 1 (unadjusted)	86.8 ± 16.7	80.5 ± 16.2	33.3 ± 17.1	0.03	0.02
Model 2	82.4 ± 26.9	73.9 ± 27.9	27.7 ± 27.8	0.03	0.03
Model 3	69.3 ± 27.3	54.9 ± 27.7	12.7 ± 28.1	0.03	0.04
**PFHxS**					
Model 1 (unadjusted)	108.7 ± 16.7	47.6 ± 16.4	47.7 ± 16.6	0.01	0.01
Model 2	100.3 ± 27.1	37.8 ± 27.2	39.1 ± 28.3	0.02	0.02
Model 3	81.5 ± 27.5	27.9 ± 27.1	24.6 ± 28.5	0.03	0.04
**PFNA**					
Model 1 (unadjusted)	88.6 ± 16.9	78.3 ± 15.8	33.0 ± 17.3	0.02	0.003
Model 2	83.8 ± 27.5	76.7 ± 27.3	27.9 ± 27.4	0.03	0.004
Model 3	73.7 ± 27.6	53.3 ± 27.6	14.1 ± 27.7	0.02	0.002
**PFDA**					
Model 1 (unadjusted)	91.5 ± 16.4	63.5 ± 16.3	45.7 ± 17.4	0.05	0.06
Model 2	88.6 ± 27.7	61.8 ± 27.4	40.3 ± 27.2	0.05	0.07
Model 3	66.5 ± 28.2	55.0 ± 27.2	23.1 ± 27.6	0.09	0.08

Model 1, unadjusted; Model 2, adjusted for age, sex, race, baseline RMR, education, smoking status, alcohol consumption, physical activity, and dietary intervention group; Model 3, further adjusted for baseline free T3 and free T4 levels.

^a^Data are least-square means ± standard errors calculated from general linear models.

^b^PFAS levels were log_10_-transformed before analysis.

PFAS, perfluoroalkyl substance; PFDA, perfluorodecanoic acid; PFHxS, perfluorohexanesulfonic acid; PFNA, perfluorononanoic acid; PFOA, perfluorooctanoic acid; PFOS, perfluorooctanesulfonic acid; RMR, resting metabolic rate; T3, triiodothyronine; T4, thyroxine.

### Baseline PFASs and changes in other metabolic parameters

During the weight-loss period, after multivariate adjustment including baseline levels of each metabolic parameter, plasma concentrations of PFOS, PFNA, and PFDA were inversely associated with changes in visceral fat mass (*r*_s_ ranged from −0.19 to −0.27, all *P <* 0.05), and baseline PFOA was inversely associated with changes in HDL cholesterol (*r*_s_ = −0.12, *P <* 0.01) (S5 Table). During the weight regain period, baseline PFOS, PFNA, and PFDA levels were positively associated with changes in some of the parameters, including waist circumference, insulin, and leptin (*r*_s_ ranged from 0.10 to 0.15, all *P <* 0.05), and baseline PFOA and PFHxS were associated with a greater increase in visceral fat mass (*r*_s_ = 0.30 and 0.27, respectively; both *P <* 0.05) (S5 Table). The results were largely similar when analyses were stratified by sex. In sensitivity analyses, the results did not materially change when further adjusting for study location (Boston or Baton Rouge) or participant compliance (number of sessions participants attended). The table in S1 Text shows the associations of baseline PFASs with gene expression in adipose tissue.

## Discussion

In this 2-year randomized weight-loss trial, we found that higher baseline plasma PFAS concentrations were not associated with weight loss induced by energy restriction, but were significantly associated with a greater weight regain, primarily among women, during the follow-up period between 6 and 24 months. In addition, after multivariate adjustment, higher baseline PFAS levels were significantly associated with a greater decrease in RMR during the weight-loss period and a lower increase in RMR during the weight regain period.

### Comparison with other studies

To date, evidence on the influence of PFAS exposure on body weight change and metabolic parameters has been limited and has been primarily generated from cross-sectional studies that could not establish causal relationships [30,44–47]. In addition, the causes of weight change are likely heterogeneous (including diet, physical activity, and medications) and often not well understood in observational studies. Prospective evidence linking PFAS exposure with body weight regulation was primarily from studies that examined prenatal or early life exposures to PFASs in relation to body weight later in life, and the results were somewhat mixed [21–27,48,49]. For example, in 3 birth cohort studies conducted in European populations, maternal concentrations of PFASs were significantly associated with offspring body weight and other anthropometric and metabolic traits, primarily among girls [21,23,25]. However, other studies generated inconsistent findings regarding maternal PFAS exposure and offspring BMI or obesity risk, with no sex difference [22,24,49]. In addition, recently, in the European Youth Heart Study, Domazet et al. demonstrated that higher plasma PFOS concentrations during childhood, but not adolescence, were associated with greater adiposity in adolescence and young adulthood [48].

To our knowledge, the current investigation is among the first studies in adults to evaluate the associations of PFAS exposures with changes in body weight and metabolic parameters induced during a controlled weight-loss trial. All individual PFASs were significantly associated with more weight regain in women, but not in men, which was in agreement with previous studies in which the intergenerational effects of PFASs on body weight were observed only in girls and not in boys [21,25,26]. Although the reasons for these gender-specific findings are still unclear, accumulating evidence from experimental research suggests that PFASs are able to interfere with estrogen metabolism and functionalities [12,50,51]. As potential endocrine disruptors, PFASs might reduce estradiol production and the expression of some key genes related to estrogen synthesis [12], or influence estradiol concentrations through pathways such as hepatic aromatase induction, with an initial inhibition and a later stimulation [50]. Using in vitro and in silico species comparison approaches, Benninghoff et al. reported that PFASs may interact directly with estrogen receptors, suggesting that PFASs could act as weak environmental xenoestrogens [51]. The experimental evidence implies that the detrimental effects of PFASs can be sex-specific, thus supporting the notion that women may be particularly vulnerable to obesogenic effects of PFASs. In addition, it is worth noticing that women generally have a higher percentage of body fat than men [52]. Given that fat-free mass could substantially influence RMR, the difference in body composition between men and women could result in significant differences in energy homeostasis dynamics [52].

In addition to the adverse effects of PFASs on estrogen-related pathways, animal studies suggest that PFOA and PFOS may also interfere with energy homeostasis and the endocrine system through other mechanisms [14,15,18,53], including the activation of PPARα and PPARγ [18,19], key regulators in fatty acid oxidation, differentiation and normal function of adipocytes, and glucose metabolism [20,54]. An experiment on human liver cells suggested that PFOA could alter the expression of proteins regulated by hepatocyte nuclear factor 4α [55], which is a key regulator of lipid metabolism and gluconeogenesis [56]. In addition, some animal studies have suggested that PFAS exposure might disrupt thyroid hormone homeostasis, possibly via influencing uridine diphosphoglucuronosyl transferases and type 1 deiodinase [17,57]. Of note, due to the species-specific toxicokinetics (e.g., the elimination half-lives are 3–8 years in humans and 17–30 days in mice and monkeys) and tissue distribution of PFASs [18], caution is needed when extrapolating findings from animal studies to humans. In addition, mechanisms need to be elucidated to interpret the findings that higher baseline PFASs, especially PFOS and PFNA, were associated with changes in RMR, which is a major determinant of weight maintenance, in both men and women [58,59]. Finally, whether the 5 major PFASs might have different biological mechanisms and perhaps exert additive or synergistic effects also warrants further exploration.

### Strengths and limitations of study

The primary strength of the current study is that the cause of weight changes was well characterized. Unlike previous observational studies in which reasons for weight changes were usually unknown, this weight-loss trial applied energy restriction to induce the weight changes. Moreover, repeated measurements of body weight, RMR, thyroid hormones, leptin, and other metabolic biomarkers allowed documentation of longitudinal associations between PFAS exposures and changes in these parameters during the weight-loss and weight regain periods.

Several limitations should be considered as well. First, although we included men and women with a wide range of ages (30–70 years), participants in the current study were otherwise relatively homogeneous in terms of health status and body fatness because they were selected following narrow inclusion criteria. Therefore, it is unclear whether our findings can be extrapolated to more general populations. Second, we measured only the baseline plasma PFAS concentrations. However, given the long elimination half-lives (3–8 years) of these chemicals [36] and a strong stability over time observed in our pilot study, concentrations in the blood likely reflect relatively long-term PFAS exposures. Moreover, unlike many other persistent organic pollutants, PFASs are not lipophilic, and blood concentrations are therefore not affected by changes in the size of the lipid compartment [60]. Third, we did not measure ghrelin, an orexigenic hormone regulating appetite, RMR, and other key physiological processes related to weight changes [61], and the interrelationship between PFASs and ghrelin during weight changes needs to be elucidated. Fourth, we did not apply Bonferroni correction in the analyses given the inter-correlation between the PFASs (*r*_s_ ranged from 0.4 to 0.9), and the role of multiple testing could not be entirely excluded. Fifth, physical activity was assessed using the Baecke questionnaire, which might be subject to measurement errors, although a validation study conducted in US adults has shown reasonable validity of this questionnaire [62]. In addition, although some covariates including education, smoking status, and physical activity were adjusted for in our study, we could not entirely exclude the possibility that unmeasured or residual confounding by socioeconomic and psychosocial factors, as well as participants’ usual diet, might partially account for the associations we observed. One particular concern is that PFASs are extensively used in food packaging due to their oil- and water-repellant characteristics [32]. If some participants relapsed to their usual pre-randomization diet and this diet was rich in foods that are contaminated by PFASs through food packaging and are also dense in energy, they might thus have gained weight faster. However, when we further controlled for the frequency of craving hamburgers, French fries, or donuts at baseline assessed using a questionnaire, the results were largely unchanged. In addition, humans are exposed to PFASs through multiple pathways, including drinking water and contaminated seafood [31], although these factors are not established risk factors for weight gain. Moreover, we adjusted for the number of study sessions that participants attended, which is a measurement of compliance to the prescribed diet. Finally, lipophilic persistent pollutants with obesogenic effects (such as hexachlorobenzene [HCB] and dichlorodiphenyldichloroethylene [DDE]) might have confounded the associations of PFASs with changes in body weight and RMR. However, in 793 women participating in the Nurses’ Health Study II, weak associations were observed between PFASs and lipophilic persistent pollutants (e.g., the *r*_s_ of PFOA and PFOS with HCB was 0.07 and 0.06, respectively, and the *r*_s_ of PFOA and PFOS with DDE was 0.05 and 0.06, respectively), suggesting that confounding by these pollutants would not be substantial.

### Implications of findings

Our study provides the first piece of evidence from a controlled weight-loss trial that higher baseline plasma PFAS concentrations in adults are associated with a greater weight regain, especially in women, possibly due to suppressed RMR levels. These findings imply that overweight and obese individuals with relatively low PFAS exposures might potentially benefit more from weight-loss interventions. Although the production of PFOS and PFOA in the US has largely been phased out [31,63], the production of other PFASs, such as PFNA, may continue or even increase, especially in developing countries [64]. Given the persistence of these PFASs in the environment and the human body, their potential adverse effects remain a public health concern.

### Conclusions

In a diet-induced weight-loss setting among overweight and obese individuals, higher baseline plasma PFAS concentrations were significantly associated with greater weight regain, especially in women, accompanied by a slower regression of RMR. These findings suggest that environmental chemicals may play a role in the current obesity epidemic. More studies are warranted to elucidate the mechanisms underlying the link between PFAS exposure and weight regulation in humans.

## Supporting information

S1 Fig Trajectory of changes in body weight of all participants according to tertiles of PFAS concentrations. Data are least-square means, adjusted for age, sex, race, education, smoking, alcohol consumption, physical activity, menopausal status (women only), hormone replacement therapy (women only), dietary intervention group, baseline free T3 and free T4 levels, and baseline body weight.
(TIF)

S1 Table Comparisons of characteristics between included and excluded participants. (DOCX)

S2 Table Partial Spearman correlation coefficients between baseline PFAS concentrations and baseline metabolic parameters. (DOCX)

S3 Table Changes in body weight (6–24 months) according to baseline plasma PFAS concentrations among women (*n =* 318). (DOCX)

S4 Table Sex-stratified analyses of changes in RMR according to baseline PFAS concentrations. (DOCX)

S5 Table Partial Spearman correlation coefficients between baseline PFAS concentrations and changes in other metabolic parameters. (DOCX)

S1 Text Gene expression profiling of adipose tissue. (DOCX)

S2 Text STROBE checklist. (DOCX)

S3 Text Study protocol and analysis plan. (PDF)

## Abbreviations

BMI: body mass index
HDL: high-density lipoprotein
HOMA-IR: homeostatic model assessment of insulin resistance
LDL: low-density lipoprotein
PFAS: perfluoroalkyl substance
PFDA: perfluorodecanoic acid
PFHxS: perfluorohexanesulfonic acid
PFNA: perfluorononanoic acid
PFOA: perfluorooctanoic acid
PFOS: perfluorooctanesulfonic acid
PPAR: peroxisome proliferator-activated receptor
RMR: resting metabolic rate
T3: triiodothyronine
T4: thyroxine
