# Supplementary figures and images for: Perfluoroalkyl substances and changes in body weight and resting metabolic rate in response to weight-loss diets: A prospective study
Source: PLoS Med. 2018 Feb 13;15(2):e1002502. doi: 10.1371/journal.pmed.1002502 (PMC5810983; doi:10.1371/journal.pmed.1002502)

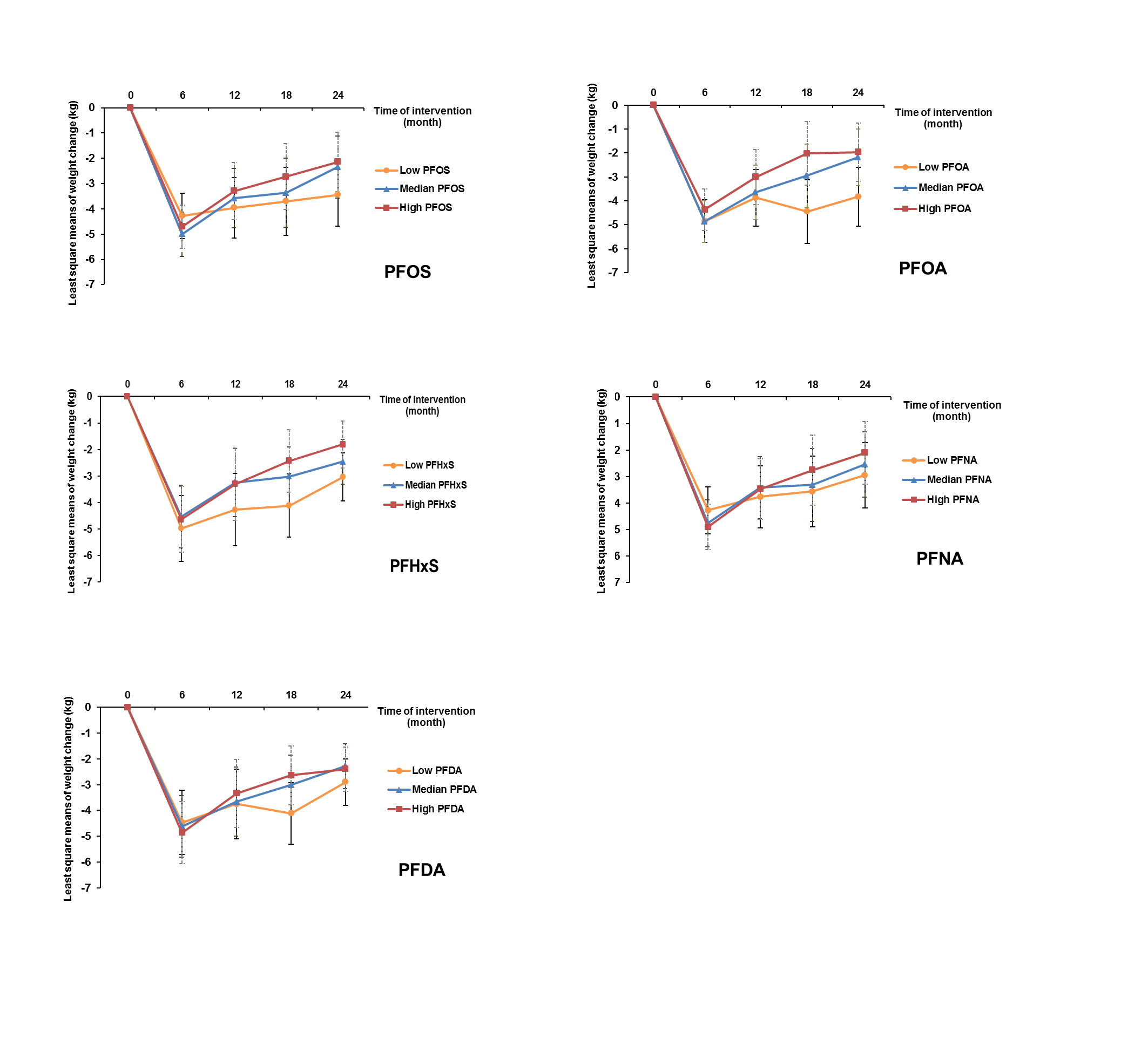

Supplement: S1 Fig — Data are least-square means, adjusted for age, sex, race, education, smoking, alcohol consumption, physical activity, menopausal status (women only), hormone replacement therapy (women only), dietary intervention group, baseline free T3 and free T4 levels, and baseline body weight. (TIF) [file pmed.1002502.s001.tif]
