# Supplementary material for: Perfluoroalkyl substances and changes in body weight and resting metabolic rate in response to weight-loss diets: A prospective study
Source: PLoS Med. 2018 Feb 13;15(2):e1002502. doi: 10.1371/journal.pmed.1002502 (PMC5810983; doi:10.1371/journal.pmed.1002502)
Supplement: S1 Table — (DOCX) [file pmed.1002502.s002.docx]

**S1 Table**. **Comparisons of characteristics between included and excluded participants**.

|  | **Included (n=621)** | **Non-included (n=190)** | ***P*** |
| --- | --- | --- | --- |
| Age (years) | 51.4 ± 9.1 | 49.1 ± 9.5 | 0.01 |
| Sex, men, % | 38.2 | 31.1 | 0.10 |
| Race, White, % | 80.5 | 75.3 | 0.10 |
| BMI (kg/m^2^) | 32.6 ± 3.8 | 33.1 ± 3.9 | 0.12 |
| Weight (kg) | 92.7 ± 15.5 | 93.4 ± 15.7 | 0.57 |
| Waist circumference (cm) | 103.3 ± 13.1 | 103.6 ± 12.9 | 0.79 |
| Resting metabolic rate (kcal/24h) | 1548.0 ± 300 | 1550.0 ± 300 | 0.94 |
| Education level, some college, % | 20.9 | 26.3 | 0.26 |
| Current smoker, yes, % | 3.5 | 4.7 | 0.72 |
| Alcohol consumption (drinks/week) | 2.2 ± 3.0 | 1.9 ± 2.7 | 0.16 |
| Physical activity ^a^ | 1.58 ± 0.1 | 1.59 ± 0.1 | 0.41 |
| Systolic blood pressure (mmHg) | 119.7 ± 13.5 | 118.7 ± 13.0 | 0.40 |
| Diastolic blood pressure (mmHg) | 75.4 ± 9.2 | 74.9 ± 10.0 | 0.53 |
| Glucose (mg/dL) | 91.9 ± 12.1 | 92.0 ± 17.8 | 0.95 |
| Insulin (µU/mL) | 12.3 ± 7.9 | 11.7 ± 6.8 | 0.35 |
| Total cholesterol (mg/dL) | 250.8 ± 106.4 | 250.5 ± 106.9 | 0.97 |
| LDL cholesterol (mg/dL) | 125.4 ± 32.2 | 126.9 ± 31.0 | 0.57 |
| HDL cholesterol (mg/dL) | 48.9 ± 13.7 | 49.9 ± 16.0 | 0.40 |
| Triglycerides (mg/dL) | 143.4 ± 85.7 | 135.9 ± 82.5 | 0.29 |

^a^ Physical activity estimated by the Baecke Questionnaire.
