# Supplementary material for: Perfluoroalkyl substances and changes in body weight and resting metabolic rate in response to weight-loss diets: A prospective study
Source: PLoS Med. 2018 Feb 13;15(2):e1002502. doi: 10.1371/journal.pmed.1002502 (PMC5810983; doi:10.1371/journal.pmed.1002502)
Supplement: S2 Table — (DOCX) [file pmed.1002502.s003.docx]

**S2 Table**. **Partial Spearman correlation coefficients among baseline PFASs and baseline metabolic parameters.**

|  | **N** | **PFOS** | **PFOA** | **PFHxS** | **PFNA** | **PFDA** |
| --- | --- | --- | --- | --- | --- | --- |
| PFOA | 621 | **0.63***** |  |  |  |  |
| PFHxS | 621 | **0.54***** | **0.50***** |  |  |  |
| PFNA | 621 | **0.79***** | **0.56***** | **0.47***** |  |  |
| PFDA | 621 | **0.71***** | **0.47***** | **0.38***** | **0.85***** | **1.00** |
| Body weight | 621 | -0.02 | 0.05 | -0.003 | 0.01 | -0.05 |
| Waist circumference | 621 | -0.01 | 0.07 | 0.04 | 0.03 | -0.04 |
| BMI | 621 | -0.01 | 0.04 | 0.04 | 0.004 | -0.06 |
| Resting metabolic rate | 620 | -0.01 | 0.03 | 0.02 | 0.05 | -0.02 |
| Whole body fat (%) | 330 | 0.001 | 0.09 | -0.01 | 0.04 | -0.02 |
| VAT fat mass | 150 | 0.10 | 0.13 | 0.13 | **0.24**** | **0.17*** |
| Hepatic fat mass | 150 | 0.11 | 0.12 | 0.09 | 0.06 | 0.13 |
| Systolic blood pressure | 621 | 0.07 | 0.04 | 0.03 | **0.09*** | 0.01 |
| Diastolic blood pressure | 621 | **0.15***** | **0.10*** | **0.09*** | **0.18***** | 0.06 |
| Glucose | 621 | 0.08 | 0.05 | 0.04 | **0.15***** | 0.08 |
| Insulin | 615 | **0.10*** | **0.10*** | 0.07 | **0.14***** | 0.04 |
| HbA1C | 620 | -0.01 | -0.03 | -0.03 | 0.01 | 0.02 |
| HOMA_IR ^a^ | 615 | **0.10*** | **0.10*** | 0.07 | **0.15***** | 0.05 |
| Triglycerides | 621 | -0.02 | **0.08*** | 0.04 | 0.003 | -0.07 |
| Total cholesterol | 621 | 0.04 | 0.02 | -0.01 | 0.04 | 0.06 |
| LDL cholesterol | 621 | **0.09*** | 0.06 | 0.04 | 0.08 | **0.11**** |
| HDL cholesterol | 621 | 0.01 | **-0.10*** | 0.002 | -0.03 | 0.03 |
| Free T3 | 531 | **0.12**** | **0.15***** | **0.11**** | **0.09*** | 0.04 |
| Total T3 | 521 | 0.06 | 0.08 | 0.07 | 0.03 | -0.01 |
| Free T4 | 552 | 0.08 | 0.06 | **0.10*** | **0.09*** | 0.06 |
| Total T4 | 519 | **0.10*** | 0.03 | **0.10*** | 0.07 | 0.03 |
| TSH | 550 | -0.01 | -0.03 | -0.02 | -0.04 | -0.03 |
| Leptin | 561 | 0.05 | **0.09*** | 0.08 | 0.06 | 0.01 |
| Leptin soluble receptor | 562 | 0.08 | 0.07 | 0.06 | 0.04 | 0.001 |

Values are adjusted for age, sex, race, education, smoking status, alcohol consumption, physical activity, menopausal status (women only), hormone replacement therapy (women only), and dietary intervention groups.

^a^ HOMA-IR was calculated using the updated HOMA model (HOMA2) described by Levy et al.

****P*<0.001 ***P*<0.01 **P*<0.05.
