# Supplementary material for: Perfluoroalkyl substances and changes in body weight and resting metabolic rate in response to weight-loss diets: A prospective study
Source: PLoS Med. 2018 Feb 13;15(2):e1002502. doi: 10.1371/journal.pmed.1002502 (PMC5810983; doi:10.1371/journal.pmed.1002502)
Supplement: S3 Table — (DOCX) [file pmed.1002502.s004.docx]

**S3 Table**. **Changes in body weight (6-24 months)^a^ according to baseline plasma PFAS concentrations among women (n=318).**

|  | **Tertiles of PFAS concentrations** | | |  |  |
| --- | --- | --- | --- | --- | --- |
|  | **T1** | **T2** | **T3** | ***P* _trend_** | ***P* _continuous_** ^b^ |
| **Weight change (kg) during 6-24 months** | | | | |  |
| PFOS |  |  |  |  |  |
| Model 1 | 1.56±0.4 | 3.32±0.5 | 3.34±0.5 | 0.005 | 0.01 |
| Model 2 | 1.54±0.4 | 3.25±0.5 | 3.43±0.5 | 0.003 | 0.006 |
| Model 3 | 1.36±0.4 | 3.34±0.5 | 3.60±0.5 | 0.001 | 0.002 |
| Model 4 | 1.19±0.9 | 2.75±1.0 | 2.61±1.0 | 0.03 | 0.04 |
| Model 5 | 1.10±1.0 | 2.81±1.0 | 2.72±1.0 | 0.02 | 0.03 |
| PFOA |  |  |  |  |  |
| Model 1 | 1.75±0.4 | 2.99±0.5 | 3.52±0.6 | 0.008 | 0.01 |
| Model 2 | 1.70±0.4 | 3.04±0.5 | 3.54±0.6 | 0.006 | 0.007 |
| Model 3 | 1.55±0.4 | 3.13±0.5 | 3.67±0.6 | 0.003 | 0.002 |
| Model 4 | 1.36±0.9 | 2.62±1.0 | 2.72±1.0 | 0.04 | 0.02 |
| Model 5 | 1.36±1.0 | 2.82±1.0 | 2.91±1.1 | 0.03 | 0.01 |
| PFHxS |  |  |  |  |  |
| Model 1 | 2.03±0.4 | 2.59±0.5 | 3.86±0.6 | 0.01 | 0.04 |
| Model 2 | 1.97±0.4 | 2.60±0.5 | 3.95±0.6 | 0.008 | 0.02 |
| Model 3 | 1.83±0.4 | 2.71±0.5 | 4.11±0.6 | 0.003 | 0.01 |
| Model 4 | 1.41±1.0 | 2.09±1.0 | 3.10±1.1 | 0.02 | 0.04 |
| Model 5 | 1.39±1.0 | 2.16±1.0 | 3.27±1.1 | 0.01 | 0.03 |
| PFNA |  |  |  |  |  |
| Model 1 | 1.80±0.4 | 2.04±0.5 | 4.07±0.5 | <0.001 | <0.001 |
| Model 2 | 1.76±0.4 | 2.05±0.5 | 4.10±0.5 | <0.001 | <0.001 |
| Model 3 | 1.65±0.5 | 2.10±0.5 | 4.18±0.5 | <0.001 | <0.001 |
| Model 4 | 1.44±0.9 | 1.31±1.0 | 3.11±1.0 | 0.02 | 0.008 |
| Model 5 | 1.41±1.0 | 1.29±1.0 | 3.21±1.0 | 0.01 | 0.005 |
| PFDA |  |  |  |  |  |
| Model 1 | 1.89±0.5 | 2.40±0.5 | 3.48±0.5 | 0.02 | 0.01 |
| Model 2 | 1.81±0.5 | 2.44±0.5 | 3.51±0.5 | 0.01 | 0.009 |
| Model 3 | 1.77±0.5 | 2.45±0.5 | 3.55±0.5 | 0.009 | 0.007 |
| Model 4 | 1.44±1.0 | 1.97±1.0 | 2.66±1.0 | 0.05 | 0.03 |
| Model 5 | 1.43±1.0 | 1.96±1.0 | 2.73±1.0 | 0.04 | 0.02 |

^a^ Data are least-square means ± standard error calculated from general linear model.

^b^ PFAS levels were log_10_-transformed before analysis.

Model 1, unadjusted;

Model 2, adjusted for baseline BMI;

Model 3, further adjusted for age and dietary intervention groups;

Model 4, further adjusted for race, education, smoking status, alcohol consumption, physical activity;

Model 5, further adjusted for menopausal status and hormone replacement therapy.
