# Supplementary material for: Perfluoroalkyl substances and changes in body weight and resting metabolic rate in response to weight-loss diets: A prospective study
Source: PLoS Med. 2018 Feb 13;15(2):e1002502. doi: 10.1371/journal.pmed.1002502 (PMC5810983; doi:10.1371/journal.pmed.1002502)
Supplement: S4 Table — (DOCX) [file pmed.1002502.s005.docx]

**S4 Table. Sex-stratified analyses of changes in RMR according to baseline PFAS concentrations.**

|  |  | **Tertile Levels of PFASs** | | |  |  |
| --- | --- | --- | --- | --- | --- | --- |
|  |  | **T1** | **T2** | **T3** | ***P* _trend_** | ***P* _interaction_** |
|  | **RMR change (kcal/day) during 0-6months** | | | | |  |
| **PFOS** | Men | -11.6±31.7 | -38.3±31.3 | -41.7±29.3 | 0.32 | 0.87 |
|  | Women | -19.2±18.8 | -29.7±19.8 | -60.4±18.5 | 0.01 |  |
|  |  |  |  |  |  |  |
| **PFOA** | Men | -67.3±32.7 | -25.6±29.7 | -12.8±29.6 | 0.10 | 0.10 |
|  | Women | -34.7±18.5 | -31.0±19.4 | -51.7±19.8 | 0.38 |  |
|  |  |  |  |  |  |  |
| **PFHxS** | Men | -44.5±34.9 | -22.3±29.8 | -32.0±31.2 | 0.89 | 0.89 |
|  | Women | -34.1±18.0 | -37.0±19.1 | -52.7±20.8 | 0.32 |  |
|  |  |  |  |  |  |  |
| **PFNA** | Men | -10.2±30.5 | -33.5±30.2 | -53.7±31.0 | 0.13 | 0.57 |
|  | Women | -17.5±18.8 | -37.5±19.3 | -61.5±18.8 | 0.008 |  |
|  |  |  |  |  |  |  |
| **PFDA** | Men | -34.2±30.3 | -25.0±30.2 | -35.6±31.6 | 0.97 | 0.63 |
|  | Women | -25.4±20.0 | -24.2±19.0 | -55.8±18.1 | 0.05 |  |
|  | **RMR change (kcal/day) during 6-24 months** | | | | |  |
| **PFOS** | Men | 46.8±50.7 | 60.8±53.1 | -40.2±51.1 | 0.05 | 0.90 |
|  | Women | 141.6±33.8 | 90.1±35.9 | 47.7±32.4 | 0.001 |  |
|  |  |  |  |  |  |  |
| **PFOA** | Men | 64.9±53.7 | 25.9±50.5 | -32.3±50.9 | 0.04 | 0.22 |
|  | Women | 96.4±33.1 | 90.8±34.5 | 66.0±36.9 | 0.37 |  |
|  |  |  |  |  |  |  |
| **PFHxS** | Men | 82.1±57.7 | -9.06±50.4 | -19.1±53.5 | 0.14 | 0.51 |
|  | Women | 106.9±32.0 | 64.0±34.7 | 70.1±37.5 | 0.16 |  |
|  |  |  |  |  |  |  |
| **PFNA** | Men | 55.6±49.9 | 8.91±51.8 | -41.8±55.4 | 0.06 | 0.30 |
|  | Women | 100.4±34.2 | 100.6±34.7 | 62.6±34.4 | 0.23 |  |
|  |  |  |  |  |  |  |
| **PFDA** | Men | 36.3±49.5 | 15.9±52.4 | -17.0±56.4 | 0.29 | 0.73 |
|  | Women | 105.6±36.4 | 101.0±33.6 | 66.0±33.3 | 0.19 |  |

Data are least-square means ± standard error calculated from general linear model, with adjustment of age, race, baseline RMR, education, smoking status, alcohol consumption, physical activity, dietary intervention groups, and baseline free T3 and free T4 levels. There were 219 men and 337 women in the first 6 months, and 158 men and 235 women during the period of 6-24 months.
