# Supplementary material for: Perfluoroalkyl substances and changes in body weight and resting metabolic rate in response to weight-loss diets: A prospective study
Source: PLoS Med. 2018 Feb 13;15(2):e1002502. doi: 10.1371/journal.pmed.1002502 (PMC5810983; doi:10.1371/journal.pmed.1002502)
Supplement: S5 Table — (DOCX) [file pmed.1002502.s006.docx]

**S5 Table . Partial Spearman correlation coefficients between baseline PFAS concentrations and changes in other metabolic parameters.**

| **0-6 months** | **N** | **PFOS** | **PFOA** | **PFHxS** | **PFNA** | **PFDA** |
| --- | --- | --- | --- | --- | --- | --- |
| △Waist circumference | 617 | 0.02 | 0.02 | 0.02 | -0.02 | -0.04 |
| △Diastolic blood pressure | 615 | 0.04 | 0.07 | 0.02 | 0.01 | -0.01 |
| △Glucose | 592 | -0.02 | 0.04 | 0.02 | -0.06 | -0.08 |
| △Insulin | 570 | -0.004 | 0.04 | 0.03 | -0.03 | -0.03 |
| △Triglycerides | 592 | -0.006 | 0.03 | 0.02 | -0.005 | -0.02 |
| △LDL cholesterol | 592 | -0.04 | -0.03 | 0.02 | -0.02 | -0.02 |
| △HDL cholesterol | 592 | -0.06 | **-0.12**** | -0.04 | -0.04 | -0.06 |
| △Total fat mass | 283 | -0.02 | 0.04 | 0.03 | -0.08 | -0.06 |
| △VAT mass | 126 | **-0.19*** | -0.14 | -0.11 | **-0.27**** | **-0.26**** |
| △Free T3 | 522 | 0.006 | 0.02 | 0.01 | 0.005 | -0.003 |
| △Free T4 | 546 | -0.01 | -0.03 | 0.04 | 0.02 | 0.02 |
| △Leptin | 557 | -0.02 | -0.002 | 0.03 | -0.03 | -0.04 |
| **6-24 months** |  |  |  |  |  |  |
| △Waist circumference | 486 | **0.14**** | 0.08 | 0.08 | **0.15***** | 0.08 |
| △Diastolic blood pressure | 481 | -0.01 | -0.03 | -0.02 | 0.004 | 0.001 |
| △Glucose | 459 | 0.002 | 0.01 | -0.02 | 0.03 | 0.03 |
| △Insulin | 439 | 0.07 | 0.04 | 0.01 | **0.13**** | **0.10*** |
| △Triglycerides | 460 | -0.07 | -0.02 | -0.05 | -0.03 | -0.02 |
| △LDL cholesterol | 460 | -0.04 | 0.03 | 0.005 | -0.06 | -0.07 |
| △HDL cholesterol | 460 | 0.04 | 0.03 | 0.02 | 0.01 | -0.03 |
| △Total fat mass | 189 | -0.06 | -0.01 | -0.03 | -0.10 | -0.07 |
| △VAT mass | 89 | 0.16 | **0.30**** | **0.27*** | 0.11 | 0.06 |
| △Free T3 | 409 | -0.08 | -0.02 | -0.05 | -0.08 | -0.08 |
| △Free T4 | 411 | -0.01 | 0.002 | -0.02 | -0.03 | -0.05 |
| △Leptin | 428 | 0.04 | 0.03 | -0.01 | **0.10*** | 0.07 |

Values are adjusted for age, sex, race, education, smoking, alcohol consumption, physical activity, menopausal status (women only), hormone replacement therapy (women only), dietary intervention groups, and baseline levels of each metabolic parameter; Δ= follow-up value −baseline value. T3: triiodothyronine; T4: thyroxine.

* *P*<0.05; ** *P*<0.01; *** *P*<0.001
