# Supplementary material for: Perfluoroalkyl substances and changes in body weight and resting metabolic rate in response to weight-loss diets: A prospective study
Source: PLoS Med. 2018 Feb 13;15(2):e1002502. doi: 10.1371/journal.pmed.1002502 (PMC5810983; doi:10.1371/journal.pmed.1002502)
Supplement: S1 Text — (DOCX) [file pmed.1002502.s007.docx]

**Gene expression profiling of adipose tissue**

At baseline and 6 months, adipose tissues were obtained from 103 participants after a 12-hour overnight fast. Abdominal subcutaneous adipose tissue (approximately 500 mg) was collected using the Bergstrom technique with suction (Micrins Inc., Lake Forest, IL). Total RNA was extracted by Trizol (Invitrogen, Carlsbad, CA) followed by a clean-up on a RNA binding column (RNAEasy; Qiagen, Valencia, CA). The quantity and integrity of the RNA was analyzed by spectrophotometry and gel electrophoresis. RNA was labeled using the Illumina TotalPrep RNA amplification kit (Applied Biosystems, Carlsbad, CA). Gene expression was measured by direct hybridization using the Illumina HT-12v3 expression beadchip (Illumina, San Diego, CA). After excluding 15 individuals without PFAS assessments, data from 88 participants were included in the final analysis.

Illumina gene expression data were quantile normalized to the median distribution and then log2 transformed. The probe and sample means were centered to zero. Quality control was performed using the eQTL-mapping pipeline. Linear regression analyses were employed to examine the associations between PFAS concentrations and adipose tissue gene expression, with correction for multiple testing using a Bonferroni approach. Genes were considered significantly expressed with a threshold of *P* <1.02×10^-6^ (0.05/48803 probes). These statistical analyses were performed with SAS software (version 9.4) and R (version 3.2.2).

**S6 Table** shows the associations of baseline PFASs with gene expression in adipose tissue. ZMAT1 (Zinc Finger Matrin-Type 1), RPS17 (Ribosomal protein S17), TFG (TRK-fused gene), and HS.544194 were among the top genes for which the expression levels were associated with PFAS concentrations (*P* ranged from 1.33 ×10^-6^ to 7.26×10^-6^), although none of the associations reached genome-wide statistical significance. In a sensitivity analysis, when Westfall/Young method was used to account for multiple testing, none of the associations reached statistical significance.

**S6 Table**. Associations between plasma PFAS concentrations and gene expression in adipose tissue

| **Probe ID** | **Gene Symbol** | **Estimate** | **SE** | ***P*** | **Chromosome** |
| --- | --- | --- | --- | --- | --- |
| **PFNA** |  |  |  |  |  |
| ILMN_1738536 | ZMAT1 | -0.27 | 0.05 | 1.33 ×10^-6^ | X |
| ILMN_2341815 | TFG | 1.02 | 0.21 | 6.75×10^-6^ | 3 |
| ILMN_1912732 | HS.544194 | 0.25 | 0.05 | 7.26×10^-6^ |  |
| ILMN_1898451 | HS.539065 | -0.21 | 0.05 | 1.63×10^-5^ | 11 |
| ILMN_1871488 | HS.542920 | -0.20 | 0.04 | 2.23×10^-5^ | 3 |
| ILMN_1880052 | HS.575583 | 0.31 | 0.07 | 3.32×10^-5^ | 8 |
| ILMN_1664776 | EFR3A | -0.31 | 0.07 | 3.66×10^-5^ | 8 |
| **PFOA** |  |  |  |  |  |
| ILMN_1898451 | HS.539065 | -0.25 | 0.05 | 2.08×10^-6^ | 11 |
| ILMN_1871488 | HS.542920 | -0.21 | 0.05 | 2.96×10^-5^ | 3 |
| **PFOS** |  |  |  |  |  |
| ILMN_2207533 | RPS17 | 0.26 | 0.05 | 5.66×10^-6^ | 15 |
| ILMN_2341815 | TFG | 0.93 | 0.21 | 3.46×10^-5^ | 3 |
| ILMN_1664776 | EFR3A | -0.31 | 0.07 | 3.49×10^-5^ | 8 |
| ILMN_1662370 | LOC646906 | 0.24 | 0.06 | 7.24×10^-5^ | 19 |
| ILMN_1738536 | ZMAT1 | -0.22 | 0.05 | 7.28×10^-5^ | X |
| **PFDA** |  |  |  |  |  |
| ILMN_1670746 | BOLL | -0.21 | 0.05 | 4.33×10^-5^ | 2 |
| ILMN_1912732 | HS.544194 | 0.25 | 0.06 | 6.85×10^-5^ |  |
| ILMN_1840923 | HS.145181 | -0.23 | 0.05 | 7.36×10^-5^ | 5 |
| ILMN_1853908 | HS.334403 | -0.18 | 0.04 | 9.61×10^-5^ | 3 |
| **PFHxS** |  |  |  |  |  |
| ILMN_1662370 | LOC646906 | 0.23 | 0.05 | 1.86×10^-5^ | 19 |
| ILMN_1871488 | HS.542920 | -0.16 | 0.04 | 9.31×10^-5^ | 3 |

Values are adjusted for age, sex, smoking status, and technical covariates.
