# Supplementary material for: Perfluoroalkyl substances and changes in body weight and resting metabolic rate in response to weight-loss diets: A prospective study
Source: PLoS Med. 2018 Feb 13;15(2):e1002502. doi: 10.1371/journal.pmed.1002502 (PMC5810983; doi:10.1371/journal.pmed.1002502)
Supplement: S3 Text — (PDF) [file pmed.1002502.s009.pdf]

Perfluoroalkyl Substances and Changes in Body Weight and Resting Metabolic Rate in The POUNDS Lost Trial

Overview of Study Design

The POUNDS LOST Trial is a randomized, double-blinded clinical trial that examined the effects of four calorie-restricted diets on weight loss. The four diet groups were: 1) Low-fat, average-protein (20% fat, 15% protein, 65% carbohydrate, 2) Low-fat, high-protein (20% fat, 25% protein, 55% carbohydrate), 3) High-fat, average-protein (40% fat, 15% protein, 45% carbohydrate), and 4) High-fat, high-protein (40% fat, 25% protein, 35% carbohydrate). Moreover, diet prescription for each participant represented a 750 kcal/day deficit from estimated energy needs, which were calculated based on measured resting energy expenditure. To facilitate blinding, the diets used similar foods in different proportions. Participants, investigators, and staff were blind to diet assignment throughout the intervention. The duration of intervention was 2 years, based on the consideration that in clinical trials weight loss is typically the greatest within 6-12 months after the initiation of dietary interventions and is followed by a steady weight regain. The details have been documented previously (*N Engl J Med* 2009;360:859-73).

Participant Flow

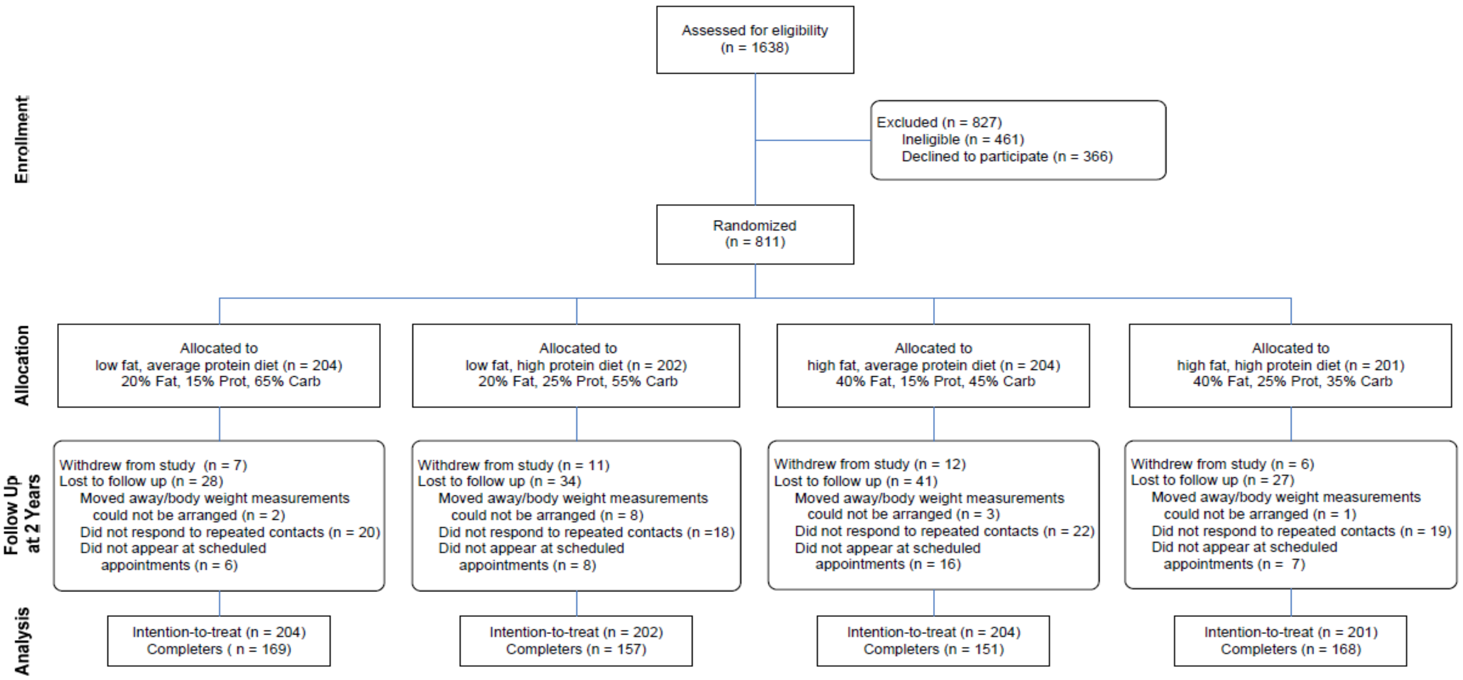

<u>Specific Aims</u> (07/01/2014)

In this proposed project, we will examine effects of PFASs on weight change among 811 men and women who participated in the well-designed and rigorously-conducted Prevention of Obesity Using Novel Dietary Strategies (POUNDS) LOST trial. This two-year trial examined the effects of reduced-calorie diets on weight loss and showed that reduced calories, rather than macronutrient compositions, led to weight loss and maintenance. Rich resources in this unique study, including repeated blood samples and data on measured body weight, waist circumference, body fat composition, biomarkers, diet, and gene expression in adipose tissue, will enable us to evaluate the effects of PFASs on body weight in a comprehensive and efficient manner.

Specifically, we will examine the following specific aims:

1. To determine the relationship between PFASs and weight change

<u>Hypothesis 1.1</u>: Higher plasma concentrations of PFOA and PFOS at baseline are associated with less weight loss at 6 months since baseline and with greater weight regain between 6 months and 2 years.

<u>Hypothesis 1.2</u>: Higher plasma concentrations of PFOA and PFOS at baseline are associated with less loss and greater regain of total fat mass, visceral fat, waist circumference, and hepatic fat.

2. To determine PFASs in relation to biological factors involved in body weight regulation

<u>Hypothesis 2.1</u>: Higher plasma concentrations of PFOA and PFOS at baseline are associated with less favorable changes in adipokines and related factors (leptin, soluble leptin receptor, adiponectin, and retinol-binding protein-4) and thyroid hormones (thyroid-stimulating hormone, total and free triiodothyronine [T3], and total and free thyroxine [T4]) during weight loss and weight regain.

3. To determine PFASs in relation to gene expression profiles in adipose tissue

<u>Hypothesis 3.1</u>: Higher plasma concentrations of PFOA and PFOS are associated with altered gene expression profiles at baseline that are consistent with PFASs' detrimental effects on energy metabolism.

<u>Secondary Aims</u>: We will explore 1) the association between PFASs and changes in blood lipids, 2) demographic, lifestyle, and dietary predictors of PFAS concentrations, and 3) effect modifications by dietary interventions, age, ethnicity, gender, and genetics on the association between PFASs and weight change.

The POUNDS LOST Trial provides an unparalleled opportunity not only for us to evaluate the effects of PFAS exposures on weight change but also to help shed light on potential pathways in a prospective manner. This prospective study has the potential to further our understanding of the role of PFASs in the etiology of adiposity in humans. The unique focus on weight loss and weight regain in a clinical trial setting will help provide direct evidence on whether PFASs should be targeted as part of a comprehensive obesity prevention and intervention strategy.

APPROACH

Participants recruitment, screening, baseline measurements, and randomization. The details have been documented previously (*N Engl J Med* 2009;360:859-73). Briefly, people who responded to recruitment were interviewed by phone to describe the study and to ascertain eligibility. Those interested and potentially eligible attended two screening visits at the clinical sites. During the first screening visit, informed consent was obtained. There were measurements of height, weight, blood pressure, urinary microalbumin, and TSH. Eligible participants were given a 5-day food diary to complete at home, and a pedometer to measure activity for 7 days. Participants attended a second screening visit 7-28 days later. Baseline nutrient intake was determined from the 5-day diet records. Other baseline measurements were obtained after the screening visits. Randomization assignments to one of 4 diet groups were generated by the data manager at the coordinating center, upon request of a study dietitian, after confirming, by computer program, that all screening activities had occurred, that the participant met all eligibility criteria, and that all required baseline data had been collected. Diet group assignments were stratified by site with varying block sizes to ensure a balance at each site. **Implementation.** After a participant was randomized, the data manager contacted the assigned dietitian to schedule the first individual visit consisting of an orientation and counseling session on the assigned diet. **Dietary teaching**. The participants were encouraged to attend all group sessions which were held 3 out of 4 weeks during the first 6 months, and 2 out of 4 weeks during 6 to 24 months; and individual sessions held every 8 weeks for the entire 24 months. Structured meal plans were provided based on the American Dietetic Association (ADA) exchange system. Daily meal plans in 2-week blocks were given to the participants. The participants were taught to follow the meal plans exactly so that they could achieve the nutrient goals. **Anthropometric Measurements and Other Body Fatness Assessments.** All measurements were performed in the morning before breakfast, and participants were instructed to urinate and wear a hospital gown before taking these measurements. **Body Weight** was measured using calibrated hospital scales on two nonconsecutive days at baseline, 6 and 24 months. **Blood and Urine Sample Collection in POUNDS LOST Trial**. Fasting blood samples and 24-hour urine samples were collected at baseline and at 6 and 24 months. **Measurement of Plasma PFASs.** PFASs were analyzed in plasma by a sensitive and reliable method based on on-line solid phase extraction (SPE) and

liquid chromatography (LC) coupled to a triple quadropole mass spectrometer (MS/MS) as developed in Norway, with minor modifications. **Measurement of Thyroid Hormones.** TSH, total and free T4, and total and free T3 in plasma were measured by a competitive electrochemiluminescence immunoassay on the Roche E Modular system (Roche Diagnostics, Indianapolis, IN). **Adipose Tissue Biopsy, RNA Extraction, and Gene Expression**. Gene expression was measured by direct hybridization using the Illumina HT-12v3 expression beadchip (Illumina, San Diego, CA).

Statistical Analysis

To ensure the validity of study results, we will restrict our analysis to 714 participants who completed 6-month follow-up for aims related to weight-loss, and 645 participants who finished the 2-year follow-up for aims related to weight regain. Among these participants, body weight and other body fatness indices were measured, and no imputed data will be included. Before a statistical analysis is conducted, we will perform preliminary data processing, which includes univariate distribution assessments for all PFAS levels and all other continuous variables using density estimation (average shifted histograms or log-spline models) and outlier detection (generalized extreme studentized deviates). Non-parametric multivariate summaries will be obtained as well (e.g., by two-dimensional average-shifted histograms). All reported P values will be two-sided. Statistical analyses will be performed primarily using SAS 9.3 (SAS Institute Inc., Cary, NC).

1. Linear regression analyses for examining PFAS levels in relation to weight loss, loss of body fat, reduction of waist circumference, and change in biomarkers between baseline and 6 months. We will first calculate Pearson correlation coefficients to determine the strength of correlations of PFAS concentrations with weight loss, as well as changes in body fat content, adipokines, thyroid hormones, and blood lipids. To minimize the influence of outliers and to detect any non-linear associations, as a secondary analysis we will use multivariate linear regression (SAS PROC GLM) to examine the linear trend of the biomarkers across quintiles of PFAS concentrations. Weight loss or other study outcomes will be entered into the model as dependent variables; quintiles of PFAS concentrations as well as study sites, age, gender, ethnicity, education levels, household income, marital status, menopause status (women only), post-menopausal hormone use (women only), smoking status, dietary intervention arms, alcohol consumption, BMI, physical activity, physical and mental status, and REE at baseline, plus compliance (number of sessions attended through 6 months), will be entered as independent variables. Least-square means of dependent variables will be calculated for each quintile of PFAS concentrations. Robust estimators of variance for these means will be calculated to allow for deviance from the assumption of normally distributed dependent variables. P values for linear

trend will be estimated by entering the median value of each quintile of PFAS levels into the model as a continuous variable.

We will run a series of stepwise models to explore the proportion of variance in weight loss that is explained by compliance, obesity risk factors, and PFASs. We will first examine the variance explained by compliance, and then will explore the proportion of remained variance that is explained by obesity risk factors and the BMI-predicting genetic score. Finally, we will examine whether PFASs explain a significant proportion of the remaining variance not explained by these aforementioned factors. The proportion explained by a factor(s) is measured by r2, which is the difference of model sum of squares between models with and without the factor(s) divided by the error sum of squares of the model without the factor(s).

Because blood samples were collected only at baseline and at 6 and 24 months, we will use the same method to examine baseline PFAS concentrations in relation to changes of adipokines, thyroid hormones, and blood lipids assessed between 6 months and 2 years, when most participants regained weight. For the same reason, this method will be applied for analysis examining baseline PFAS concentrations in relation to change of body fat mass, visceral fat, and hepatic fat between 6 months and 24 months.

2. Linear mixed model analyses for modeling PFAS levels in relation to change in weight and waist circumference between 6 and 24 months. We will use linear mixed model analyses (SAS PROC MIXED) to model baseline PCB concentrations in relation to the change of body weight between 6 and 24 months. We will investigate whether more complex correlation structures are necessary as well as use a robust empirical variance estimator that yields valid inferences even in the presence of a misspecified covariance structure for the longitudinal response. In this model, we are able to examine whether PFASs are associated with body weight and whether the rate of weight change over 18-month (6 to 24 months) follow-up differs by PFAS levels. In these analyses, we will adjust for study sites, age, gender, ethnicity, education levels, household income, marital status, smoking status, dietary intervention arms, and alcohol consumption at the trial baseline, as well as menopause status (women only), post-menopausal hormone use (women only), BMI, physical activity, physical and mental status, and REE at 6 months, plus compliance (number of sessions attended between 6 and 24 months). We will investigate the appropriateness of the linearity assumption both on time and on PFAS concentrations by considering higher-order parametric functions of time (and the corresponding interactions with PFAS levels) as well as exposure categorized into a binary variable using the median as the cutpoint.

3. Analysis plan for evaluating the association of PFASs with gene expression profile in adipose tissue. Adipose tissue gene expression data will first be adjusted for known technical and clinical covariates, as well as unknown latent variables using surrogate variable analysis. Then, these data will be assessed on the gene-level and gene set-level for association with PFAS exposures using approaches similar to those described above with correction for multiple testing.

References

1. Grun F, Blumberg B. Endocrine disrupters as obesogens. Mol Cell Endocrinol. 2009;304:19-29

2. Holtcamp W. Obesogens: An environmental link to obesity. Environ Health Perspect. 2012;120:a62-68

3. Sacks FM, Bray GA, Carey VJ, Smith SR, Ryan DH, Anton SD, McManus K, Champagne CM, Bishop LM, Laranjo N, Leboff MS, Rood JC, de Jonge L, Greenway FL, Loria CM, Obarzanek E, Williamson DA. Comparison of weight-loss diets with different compositions of fat, protein, and carbohydrates. N Engl J Med. 2009;360:859-873

4. Flegal Km CMDKBKOCL. Prevalence of obesity and trends in the distribution of body mass index among us adults, 1999-2010. JAMA: The Journal of the American Medical Association. 2012;307:491-497

5. Finkelstein EA, Trogdon JG, Cohen JW, Dietz W. Annual medical spending attributable to obesity: Payer-and service-specific estimates. Health Aff (Millwood). 2009;28:w822-831

6. Hu FB. Obesity epidemiology. New York, NY: Oxford University Press; 2008.

7. Lau C, Anitole K, Hodes C, Lai D, Pfahles-Hutchens A, Seed J. Perfluoroalkyl acids: A review of monitoring and toxicological findings. Toxicol Sci. 2007;99:366-394

8. Renner R. Growing concern over perfluorinated chemicals. Environ Sci Technol. 2001;35:154A-160A

9. Lau C, Butenhoff JL, Rogers JM. The developmental toxicity of perfluoroalkyl acids and their derivatives. Toxicol Appl Pharmacol. 2004;198:231-241

10. Zushi Y, Hogarh JN, Masunaga S. Progress and perspective of perfluorinated compound risk assessment and management in various countries and institutes. Clean Technologies and Environmental Policy. 2012;14:9-20

11. Lindstrom AB, Strynar MJ, Libelo EL. Polyfluorinated compounds: Past, present, and future. Environ Sci Technol. 2011;45:7954-7961

12. Fromme H, Tittlemier SA, Volkel W, Wilhelm M, Twardella D. Perfluorinated compounds--exposure assessment for the general population in western countries. Int J Hyg Environ Health. 2009;212:239-270

13. D'Hollander W, de Voogt P, De Coen W, Bervoets L. Perfluorinated substances in human food and other sources of human exposure. Rev Environ Contam Toxicol. 2010;208:179-215

14. Kato K, Wong LY, Jia LT, Kuklenyik Z, Calafat AM. Trends in exposure to polyfluoroalkyl chemicals in the u.S. Population: 1999-2008. Environ Sci Technol. 2011;45:8037-8045

15. Calafat AM, Wong LY, Kuklenyik Z, Reidy JA, Needham LL. Polyfluoroalkyl chemicals in the u.S. Population: Data from the national health and nutrition examination survey (nhanes) 2003-2004 and comparisons with nhanes 1999-2000. Environ Health Perspect. 2007;115:1596-1602

16. Olsen GW, Burris JM, Ehresman DJ, Froehlich JW, Seacat AM, Butenhoff JL, Zobel LR. Half-life of serum elimination of perfluorooctanesulfonate,perfluorohexanesulfonate, and perfluorooctanoate in retired fluorochemical production workers. Environ Health Perspect. 2007;115:1298-1305

17. Bartell SM, Calafat AM, Lyu C, Kato K, Ryan PB, Steenland K. Rate of decline in serum pfoa concentrations after granular activated carbon filtration at two public water systems in ohio and west virginia. Environ Health Perspect. 2010;118:222-228

18. Brede E, Wilhelm M, Goen T, Muller J, Rauchfuss K, Kraft M, Holzer J. Two-year follow-up biomonitoring pilot study of residents' and controls' PFAS plasma levels after pfoa reduction in public water system in arnsberg, germany. Int J Hyg Environ Health. 2010;213:217-223

19. Post GB, Cohn PD, Cooper KR. Perfluorooctanoic acid (pfoa), an emerging drinking water contaminant: A critical review of recent literature. Environ Res. 2012;116:93-117

20. Goldenthal EI, Jessup DC, Geil RG, Mehring JS. Final report, ninety day subacute rhesus monkey toxicity study, international research and development corporation, study no. 137-090, november 10, 1978, u.S. Epa administrative record, ar226-0447. 1978

21. Seacat AM, Thomford PJ, Hansen KJ, Clemen LA, Eldridge SR, Elcombe CR, Butenhoff JL. Sub-chronic dietary toxicity of potassium perfluorooctanesulfonate in rats. Toxicology. 2003;183:117-131

22. Seacat AM, Thomford PJ, Hansen KJ, Olsen GW, Case MT, Butenhoff JL. Subchronic toxicity studies on perfluorooctanesulfonate potassium salt in cynomolgus monkeys. Toxicol Sci. 2002;68:249-264

23. Goldenthal EI, Jessup DC, Geil RG, Jefferson ND, Arceo RJ, Ruecker FA. Final report, ninety day subacute rat toxicity study on fluorad fluorochemical, fc-143, international

research and development corporation, study no. 137- 089, 3m reference no. T-3141, november 6, 1978, u.S. Epa administrative record, ar226-0441. 1978

24. Kennedy GL, Butenhoff JL, Olsen GW, O'Connor JC, Seacat AM, Perkins RG, Biegel LB, Murphy SR, Farrar DG. The toxicology of perfluorooctanoate. Crit. Rev. Toxicol. 2004;34:351-384

25. Butenhoff J, Costa G, Elcombe C, Farrar D, Hansen K, Iwai H, Jung R, Kennedy G, Jr., Lieder P, Olsen G, Thomford P. Toxicity of ammonium perfluorooctanoate in male cynomolgus monkeys after oral dosing for 6 months. Toxicol Sci. 2002;69:244-257

26. Chang SC, Thibodeaux JR, Eastvold ML, Ehresman DJ, Bjork JA, Froehlich JW, Lau C, Singh RJ, Wallace KB, Butenhoff JL. Thyroid hormone status and pituitary function in adult rats given oral doses of perfluorooctanesulfonate (pfos). Toxicology. 2008;243:330-339

27. Yu WG, Liu W, Jin YH. Effects of perfluorooctane sulfonate on rat thyroid hormone biosynthesis and metabolism. Environ Toxicol Chem. 2009;28:990-996

28. Thibodeaux JR, Hanson RG, Rogers JM, Grey BE, Barbee BD, Richards JH, Butenhoff JL, Stevenson LA, Lau C. Exposure to perfluorooctane sulfonate during pregnancy in rat and mouse. I: Maternal and prenatal evaluations. Toxicol Sci. 2003;74:369-381

29. Lau C, Thibodeaux JR, Hanson RG, Rogers JM, Grey BE, Stanton ME, Butenhoff JL, Stevenson LA. Exposure to perfluorooctane sulfonate during pregnancy in rat and mouse. Ii: Postnatal evaluation. Toxicol Sci. 2003;74:382-392

30. Luebker DJ, York RG, Hansen KJ, Moore JA, Butenhoff JL. Neonatal mortality from in utero exposure to perfluorooctanesulfonate (pfos) in sprague-dawley rats: Dose-response, and biochemical and pharamacokinetic parameters. Toxicology. 2005;215:149-169

31. Luebker DJ, Case MT, York RG, Moore JA, Hansen KJ, Butenhoff JL. Two-generation reproduction and cross-foster studies of perfluorooctanesulfonate (pfos) in rats. Toxicology. 2005;215:126-148

32. Lau C, Thibodeaux JR, Hanson RG, Narotsky MG, Rogers JM, Lindstrom AB, Strynar MJ. Effects of perfluorooctanoic acid exposure during pregnancy in the mouse. Toxicol Sci. 2006;90:510-518

33. Hines EP, White SS, Stanko JP, Gibbs-Flournoy EA, Lau C, Fenton SE. Phenotypic dichotomy following developmental exposure to perfluorooctanoic acid (pfoa) in female cd-1 mice: Low doses induce elevated serum leptin and insulin, and overweight in mid-life. Mol Cell Endocrinol. 2009;304:97-105

34. Ikeda T, Aiba K, Fukuda K, Tanaka M. The induction of peroxisome proliferation in rat liver by perfluorinated fatty acids, metabolically inert derivatives of fatty acids. J Biochem. 1985;98:475-482

35. Rosen MB, Lee JS, Ren H, Vallanat B, Liu J, Waalkes MP, Abbott BD, Lau C, Corton JC. Toxicogenomic dissection of the perfluorooctanoic acid transcript profile in mouse liver: Evidence for the involvement of nuclear receptors ppar alpha and car. Toxicol Sci. 2008;103:46-56

36. Evans RM, Barish GD, Wang YX. Ppars and the complex journey to obesity. Nat Med. 2004;10:355-361

37. Janesick A, Blumberg B. Minireview: Ppargamma as the target of obesogens. J Steroid Biochem Mol Biol. 2011;127:4-8

38. Vanden Heuvel JP, Thompson JT, Frame SR, Gillies PJ. Differential activation of nuclear receptors by perfluorinated fatty acid analogs and natural fatty acids: A comparison of human, mouse, and rat peroxisome proliferator-activated receptor-alpha, -beta, and -gamma, liver x receptor-beta, and retinoid x receptor-alpha. Toxicol Sci. 2006;92:476-489

39. Puigserver P, Wu Z, Park CW, Graves R, Wright M, Spiegelman BM. A cold-inducible coactivator of nuclear receptors linked to adaptive thermogenesis. Cell. 1998;92:829-839

40. Spiegelman BM, Puigserver P, Wu Z. Regulation of adipogenesis and energy balance by ppargamma and pgc-1. Int J Obes Relat Metab Disord. 2000;24 Suppl 4:S8-10

41. Wu Z, Puigserver P, Spiegelman BM. Transcriptional activation of adipogenesis. Curr Opin Cell Biol. 1999;11:689-694

42. Scharmach E, Buhrke T, Lichtenstein D, Lampen A. Perfluorooctanoic acid affects the activity of the hepatocyte nuclear factor 4 alpha (hnf4alpha). Toxicol Lett. 2012;212:106-112

43. Rhee J, Inoue Y, Yoon JC, Puigserver P, Fan M, Gonzalez FJ, Spiegelman BM. Regulation of hepatic fasting response by ppargamma coactivator-1alpha (pgc-1): Requirement for hepatocyte nuclear factor 4alpha in gluconeogenesis. Proc Natl Acad Sci U S A. 2003;100:4012-4017

44. Hayhurst GP, Lee YH, Lambert G, Ward JM, Gonzalez FJ. Hepatocyte nuclear factor 4alpha (nuclear receptor 2a1) is essential for maintenance of hepatic gene expression and lipid homeostasis. Mol Cell Biol. 2001;21:1393-1403

45. Ohguchi H, Tanaka T, Uchida A, Magoori K, Kudo H, Kim I, Daigo K, Sakakibara I, Okamura M, Harigae H, Sasaki T, Osborne TF, Gonzalez FJ, Hamakubo T, Kodama T, Sakai

J. Hepatocyte nuclear factor 4alpha contributes to thyroid hormone homeostasis by cooperatively regulating the type 1 iodothyronine deiodinase gene with gata4 and kruppel-like transcription factor 9. Mol Cell Biol. 2008;28:3917-3931

46. Chang SC, Noker PE, Gorman GS, Gibson SJ, Hart JA, Ehresman DJ, Butenhoff JL. Comparative pharmacokinetics of perfluorooctanesulfonate (pfos) in rats, mice, and monkeys. Reprod Toxicol. 2012;33:428-440

47. Olsen GW, Hansen KJ, Stevenson LA, Burris JM, Mandel JH. Human donor liver and serum concentrations of perfluorooctanesulfonate and other perfluorochemicals. Environ Sci Technol. 2003;37:888-891

48. Rakhshandehroo M, Hooiveld G, Muller M, Kersten S. Comparative analysis of gene regulation by the transcription factor pparalpha between mouse and human. PLoS One. 2009;4:e6796

49. Cheung C, Akiyama TE, Ward JM, Nicol CJ, Feigenbaum L, Vinson C, Gonzalez FJ. Diminished hepatocellular proliferation in mice humanized for the nuclear receptor peroxisome proliferator-activated receptor alpha. Cancer Res. 2004;64:3849-3854

50. Palmer CN, Hsu MH, Griffin KJ, Raucy JL, Johnson EF. Peroxisome proliferator activated receptor-alpha expression in human liver. Mol Pharmacol. 1998;53:14-22

51. Holden PR, Tugwood JD. Peroxisome proliferator-activated receptor alpha: Role in rodent liver cancer and species differences. J Mol Endocrinol. 1999;22:1-8

52. Nakamura T, Ito Y, Yanagiba Y, Ramdhan DH, Kono Y, Naito H, Hayashi Y, Li Y, Aoyama T, Gonzalez FJ, Nakajima T. Microgram-order ammonium perfluorooctanoate may activate mouse peroxisome proliferator-activated receptor alpha, but not human pparalpha. Toxicology. 2009;265:27-33

53. Loccisano AE, Campbell JL, Jr., Andersen ME, Clewell HJ, 3rd. Evaluation and prediction of pharmacokinetics of pfoa and pfos in the monkey and human using a pbpk model. Regul Toxicol Pharmacol. 2011;59:157-175

54. Olsen GW, Zobel LR. Assessment of lipid, hepatic, and thyroid parameters with serum perfluorooctanoate (pfoa) concentrations in fluorochemical production workers. Int Arch Occup Environ Health. 2007;81:231-246

55. Costa G, Sartori S, Consonni D. Thirty years of medical surveillance in perfluooctanoic acid production workers. J Occup Environ Med. 2009;51:364-372

56. Sakr CJ, Kreckmann KH, Green JW, Gillies PJ, Reynolds JL, Leonard RC. Cross-sectional study of lipids and liver enzymes related to a serum biomarker of exposure (ammonium perfluorooctanoate or apfo) as part of a general health survey in a cohort of occupationally exposed workers. J Occup Environ Med. 2007;49:1086-1096

57. Sakr CJ, Leonard RC, Kreckmann KH, Slade MD, Cullen MR. Longitudinal study of serum lipids and liver enzymes in workers with occupational exposure to ammonium perfluorooctanoate. J Occup Environ Med. 2007;49:872-879

58. Frisbee SJ, Shankar A, Knox SS, Steenland K, Savitz DA, Fletcher T, Ducatman AM. Perfluorooctanoic acid, perfluorooctanesulfonate, and serum lipids in children and adolescents: Results from the c8 health project. Arch Pediatr Adolesc Med. 2010;164:860-869

59. Nelson JW, Hatch EE, Webster TF. Exposure to polyfluoroalkyl chemicals and cholesterol, body weight, and insulin resistance in the general us population. Environ. Health Perspect. 2010;118:197-202

60. Steenland K, Tinker S, Frisbee S, Ducatman A, Vaccarino V. Association of perfluorooctanoic acid and perfluorooctane sulfonate with serum lipids among adults living near a chemical plant. Am J Epidemiol. 2009;170:1268-1278

61. Olsen GW, Burlew MM, Marshall JC, Burris JM, Mandel JH. Analysis of episodes of care in a perfluorooctanesulfonyl fluoride production facility. J Occup Environ Med. 2004;46:837-846

62. Emmett EA, Zhang H, Shofer FS, Freeman D, Rodway NV, Desai C, Shaw LM. Community exposure to perfluorooctanoate: Relationships between serum levels and certain health parameters. J Occup Environ Med. 2006;48:771-779

63. C8 science panel. Status report: Pfoa and adult thyroid disease in the mid-ohio valley. December 5, 2011. <http://www.C8sciencepanel.Org/pdfs/status_report_c8_and_thyroid_disease_5dec2011.Pdf>, (accessed august 30, 2012). 2011

64. Lopez-Espinosa MJ, Mondal D, Armstrong B, Bloom MS, Fletcher T. Thyroid function and perfluoroalkyl acids in children living near a chemical plant. Environ Health Perspect. 2012;120:1036-1041

65. Melzer D, Rice N, Depledge MH, Henley WE, Galloway TS. Association between serum perfluorooctanoic acid (pfoa) and thyroid disease in the u.S. National health and nutrition examination survey. Environ Health Perspect. 2010;118:686-692

66. C8 science panel. Status report: Serum pfoa and markers of thyroid function in children in the mid-ohio valley. August 11, 2011. <http://www.C8sciencepanel.Org/pdfs/status_report_c8_and_thyroid_children_17august2011.Pdf>, (accessed august 30, 2012). 2011

67. Knox SS, Jackson T, Frisbee SJ, Javins B, Ducatman AM. Perfluorocarbon exposure, gender and thyroid function in the c8 health project. J Toxicol Sci. 2011;36:403-410

68. Ji K, Kim S, Kho Y, Paek D, Sakong J, Ha J, Choi K. Serum concentrations of major perfluorinated compounds among the general population in korea: Dietary sources and potential impact on thyroid hormones. Environ. Int. 2012;45:78-85

69. Bloom MS, Kannan K, Spliethoff HM, Tao L, Aldous KM, Vena JE. Exploratory assessment of perfluorinated compounds and human thyroid function. Physiol Behav. 2010;99:240-245

70. Gilliland FD, Mandel JS. Serum perfluorooctanoic acid and hepatic enzymes, lipoproteins, and cholesterol: A study of occupationally exposed men. Am J Ind Med. 1996;29:560-568
